# Liver cancer from the perspective of single-cell sequencing: a review combined with bibliometric analysis

**DOI:** 10.1007/s00432-024-05855-7

**Published:** 2024-06-24

**Authors:** Yanwei Ji, Qi An, Xinyu Wen, Zhou Xu, Zhengyuan Xia, Zhongyuan Xia, Qinyong Hu, Shaoqing Lei

**Affiliations:** 1 Department of Anesthesiology, Renmin Hospital of Wuhan University, Wuhan, China; 2 Department of Oncology, Renmin Hospital of Wuhan University, Wuhan, China; 3 The Second Clinical Medical College, Jiangxi Medical College, Nanchang University, Jiangxi, Nanchang, China; 4 Department of Anesthesiology, Affiliated Hospital of Guangdong Medical University, Zhanjiang, China; 5 Faculty of Chinese Medicine, State Key Laboratory of Quality Research in Chinese Medicine, Macau University of Science and Technology, Taipa, Macao, China

**Keywords:** Liver cancer, Single cell sequencing, Hepatocellular carcinoma, Tumor immune microenvironment, Immunotherapy, Bibliometric analysis

## Abstract

**Background:**

Liver cancer (LC) is a prevalent malignancy and a leading cause of cancer-related mortality worldwide. Extensive research has been conducted to enhance patient outcomes and develop effective prevention strategies, ranging from molecular mechanisms to clinical interventions. Single-cell sequencing, as a novel bioanalysis technology, has significantly contributed to the understanding of the global cognition and dynamic changes in liver cancer. However, there is a lack of bibliometric analysis in this specific research area. Therefore, the objective of this study is to provide a comprehensive overview of the knowledge structure and research hotspots in the field of single-cell sequencing in liver cancer research through the use of bibliometrics.

**Method:**

Publications related to the application of single-cell sequencing technology to liver cancer research as of December 31, 2023, were searched on the web of science core collection (WoSCC) database. VOSviewers, CiteSpace, and R package “bibliometrix” were used to conduct this bibliometric analysis.

**Results:**

A total of 331 publications from 34 countries, primarily led by China and the United States, were included in this study. The research focuses on the application of single cell sequencing technology to liver cancer, and the number of related publications has been increasing year by year. The main research institutions involved in this field are Fudan University, Sun Yat-Sen University, and the Chinese Academy of Sciences. Frontiers in Immunology and Nature Communications is the most popular journal in this field, while Cell is the most frequently co-cited journal. These publications are authored by 2799 individuals, with Fan Jia and Zhou Jian having the most published papers, and Llovet Jm being the most frequently co-cited author. The use of single cell sequencing to explore the immune microenvironment of liver cancer, as well as its implications in immunotherapy and chemotherapy, remains the central focus of this field. The emerging research hotspots are characterized by keywords such as 'Gene-Expression', 'Prognosis', 'Tumor Heterogeneity', 'Immunoregulation', and 'Tumor Immune Microenvironment'.

**Conclusion:**

This is the first bibliometric study that comprehensively summarizes the research trends and developments on the application of single cell sequencing in liver cancer. The study identifies recent research frontiers and hot directions, providing a valuable reference for researchers exploring the landscape of liver cancer, understanding the composition of the immune microenvironment, and utilizing single-cell sequencing technology to guide and enhance the prognosis of liver cancer patients.

**Supplementary Information:**

The online version contains supplementary material available at 10.1007/s00432-024-05855-7.

## Introduction

Liver cancer (LC) is a prevalent malignant tumor and a major contributor to cancer-related mortality worldwide (Villanueva 2019). The primary types of liver cancer include hepatocellular carcinoma (HCC), cholangiocarcinoma, mixed-type cancer, and hemangioma. Among these, hepatocellular carcinoma accounts for more than 80% of cases. HCC originates from hepatocytes, which are the primary functional cells of the liver. It is often associated with chronic liver diseases such as viral hepatitis, alcoholic liver disease, and non-alcoholic fatty liver disease. The incidence of LC has been steadily increasing in many regions, presenting a significant public health challenge (Forner et al. 2018; Degterev et al. 2008). Gaining a comprehensive understanding of the epidemiology, risk factors, pathogenesis, and treatment options for HCC is crucial for enhancing patient outcomes and developing effective preventive strategies. Over the years, numerous studies have been conducted to explore various aspects of LC, encompassing its molecular mechanisms to clinical management (Bruix et al. 2016).

Single-cell sequencing (SCS) has emerged as a powerful tool for studying biological systems with unprecedented resolution. Unlike traditional sequencing methods that analyze bulk cell populations, single-cell sequencing enables the examination of genetic heterogeneity between individual cells. Some of the key applications of single-cell genomics include characterizing tumor heterogeneity, mapping cell lineages during development, and analyzing complex microbial communities (Tanay and Regev 2017). The utilization of single-cell genomics has provided valuable insights into diverse biological processes. For instance, scRNA-seq was employed to discover new cell types and states in pancreatic tissue (Baron et al. 2016). Additionally, other studies have utilized single-cell DNA sequencing to track mutation dynamics in cancer (Wang et al. 2014) and lineage relationships in the early embryo (Petropoulos et al. 2016).

Therefore, a comprehensive review of scientific literature on the use of single-cell sequencing technologies in liver cancer research is essential to enhance our knowledge in this field. While some studies have provided systematic reviews and insights on the application of single-cell sequencing in liver cancer, they often lack objective visual data support, leading to issues such as subjectivity and heterogeneity (Wang et al. 2024). Overcoming these challenges is crucial for a more thorough analysis, identification of research priorities, and defining innovative research directions (Wang et al. 2024). Bibliometric analysis is a quantitative method used to assess scientific output, impact, and trends within a specific research field. It involves analyzing publication patterns, citation networks, and collaboration dynamics to gain insights into the growth, development, and knowledge dissemination within that field. A bibliometric analysis can provide a comprehensive understanding of the research landscape, key contributors, emerging trends, and research gaps (Miao et al. 2018). This analysis allows for a systematic examination of the published literature, offering an overview of research output, citation impact, and collaboration patterns. By utilizing bibliometric indicators such as publication productivity, citation counts, authorship analysis, and keyword mapping, influential studies, prolific authors and institutions, collaborative networks, and emerging research themes can be identified. This paper aims to conduct a bibliometric analysis of recent publications in the field of hepatocellular carcinoma research. The analysis will identify the main contributors, and current research status, and explore research trends and future development prospects in this field.

## Method

### Search strategy

**Fig. 1:**
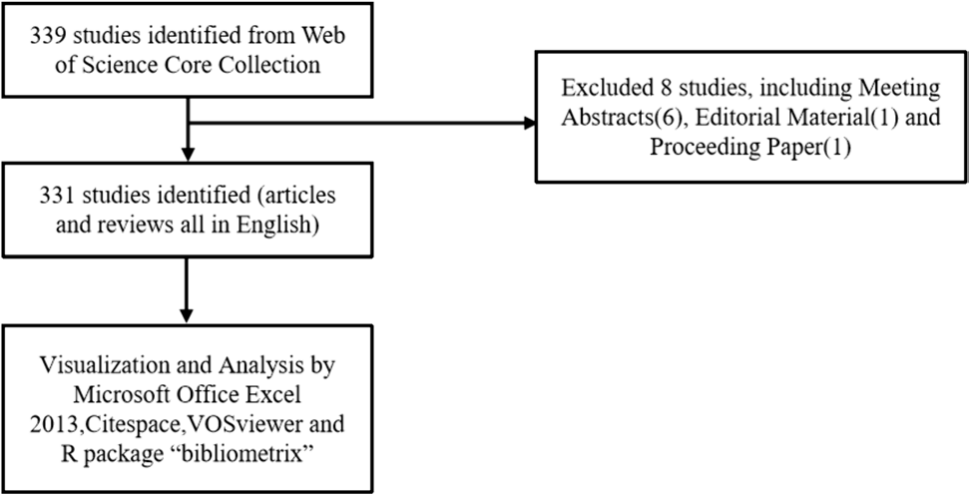
Literature search strategy and screening flow chart

The Web of Science Core Collection (WOSCC) database (https://www.webofscience.com/wos/woscc/basic-search) is a valuable resource for research publications in various fields such as natural sciences, engineering, and biomedicine. It is widely recognized and serves as a prominent source for bibliometric analysis (Birkle et al. 2020), (Ogunsakin et al. 2022), (Sheng et al. 2021). In order to explore the development trends and hot topics in the application of single cells in liver cancer research, we searched the WoSCC database using the following search formula: (TS = (single-cell sequencing)) AND TS = (liver cancer). We specifically focused on articles and reviews (including meta-analysis and systematic reviews) written in English (Fig. 1). All bibliographic records, including title, abstract, keywords, authors, institutions, addresses, journals, references, citation time, and publication year, were saved as ordinary TXT files. Our data were obtained from open databases, eliminating any ethical concerns.

### Data analysis

VOSviewer (version 1.6.19) is a bibliometric analysis software that is commonly used to extract key information from multiple publications. It is often utilized to construct collaboration, co-citation, and co-occurrence networks (Webb 1991; Yeung and Mozos 2020; Pan et al. 2018). In our study, we employed VOSviewer to conduct various analyses, including country and institution analysis, journal and co-cited journal analysis, document and co-cited document analysis, author and co-cited author analysis, and keyword co-occurrence analysis. The generated map in VOSviewer represents different projects, where nodes represent the projects. The size and color of the node indicate the quantity and classification of the item, respectively. The thickness of the lines between nodes reflects the degree of collaboration or co-citation of the projects (Wu et al. 2021), (Zhang et al. 2020).

CiteSpace (version 6.2.R4) is a software developed by Professor Chen Chaomei for bibliometric analysis and visualization (Pan et al. 2018), (Synnestvedt et al. 2005). In this study, CiteSpace was used to generate cluster analysis diagrams, peak and peak diagrams, time zone diagrams, time flows, journal double diagram overlay diagrams, etc. for journals. Additionally, citation bursts were used to analyze literature hot spots and cutting-edge trends.

The R package 'bibliometrix' (version 4.3.1) (https://www.bibliometrix.org) was utilized to construct a global distribution network of publications (Aria and Cuccurullo 2017) and the ‘H-index’ on the application of single cell sequencing in liver cancer. The H index is a hybrid quantitative indicator that can be used to assess the quantity and level of a researcher's academic output. It is defined as follows: if an individual has N papers that have been cited at least N times in their respective academic field, then their H index is N (Lin et al. 2022).

Quantitative analysis of publications was conducted using Microsoft Office Excel 2013.

The journal's impact factors and partitions are available from the Journal Citation Report (JCR).

## Result

### Analysis of annual publications

**Fig. 2:**
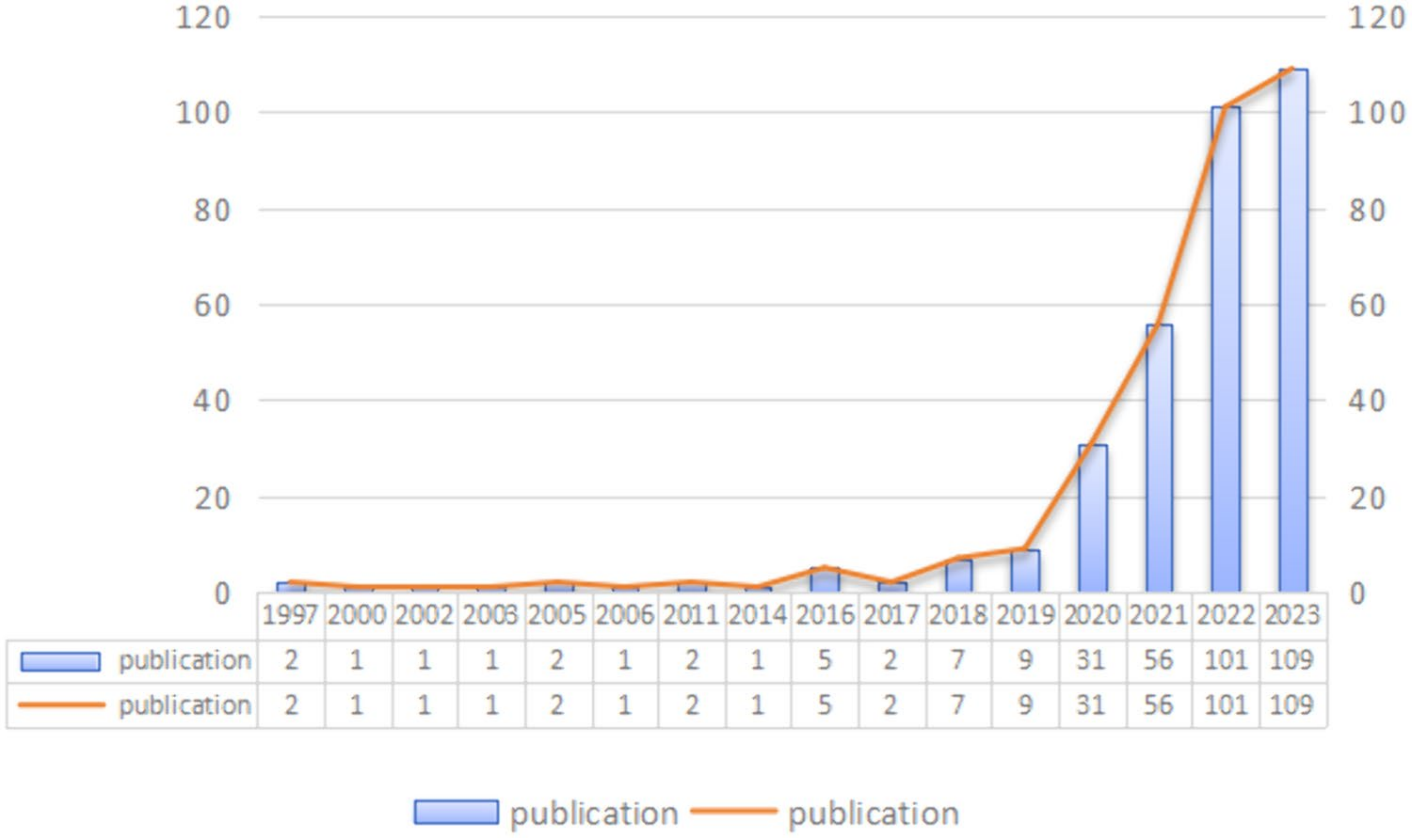
Annual output of research of application of single cell sequencing to liver cancer

According to our search strategy, a total of 331 articles were included in this study, consisting of 299 articles and 32 reviews. The development process from 1997 to the end of 2023 can be divided into three major stages. The first phase, spanning from 1997 to 2015, had an average of 0.55 publications per year, with some years having no publications. This suggests that research on the application of single cell sequencing to liver cancer was not yet conducted during this phase. The reasons could be attributed to the incomplete understanding or recognition of the relevant theoretical basis, or the imperfection and lack of promotion of the technical level. The second phase, covering the years 2016 to 2019, saw an average annual publication count of 5.75, indicating that relevant research was conducted during this period. Considering the subsequent rapid development, it can be preliminarily estimated that the maturity of relevant technologies and the establishment of an in-depth theoretical foundation may depend on the output of this stage. The third stage, mainly from 2020 to 2023, witnessed a significant increase in annual publications, reaching 74.25. Notably, the research on the application of single cell sequencing to liver cancer experienced a surge in 2022 alone, with a total of 101 scientific and technological outputs. This accounted for 30.51% of the total number of publications in this field. Therefore, it can be initially estimated that the theoretical basis of single-cell sequencing for liver cancer has been completed, and the related research techniques and methods have been recognized and popularized. However, 2023, does not exhibit the same level of growth as 2022. The total number of articles published this year was only 109, which corresponds to 32.93% of the total articles published in this field (Fig. 2).

### Country and institutional analysis

**Fig. 3:**
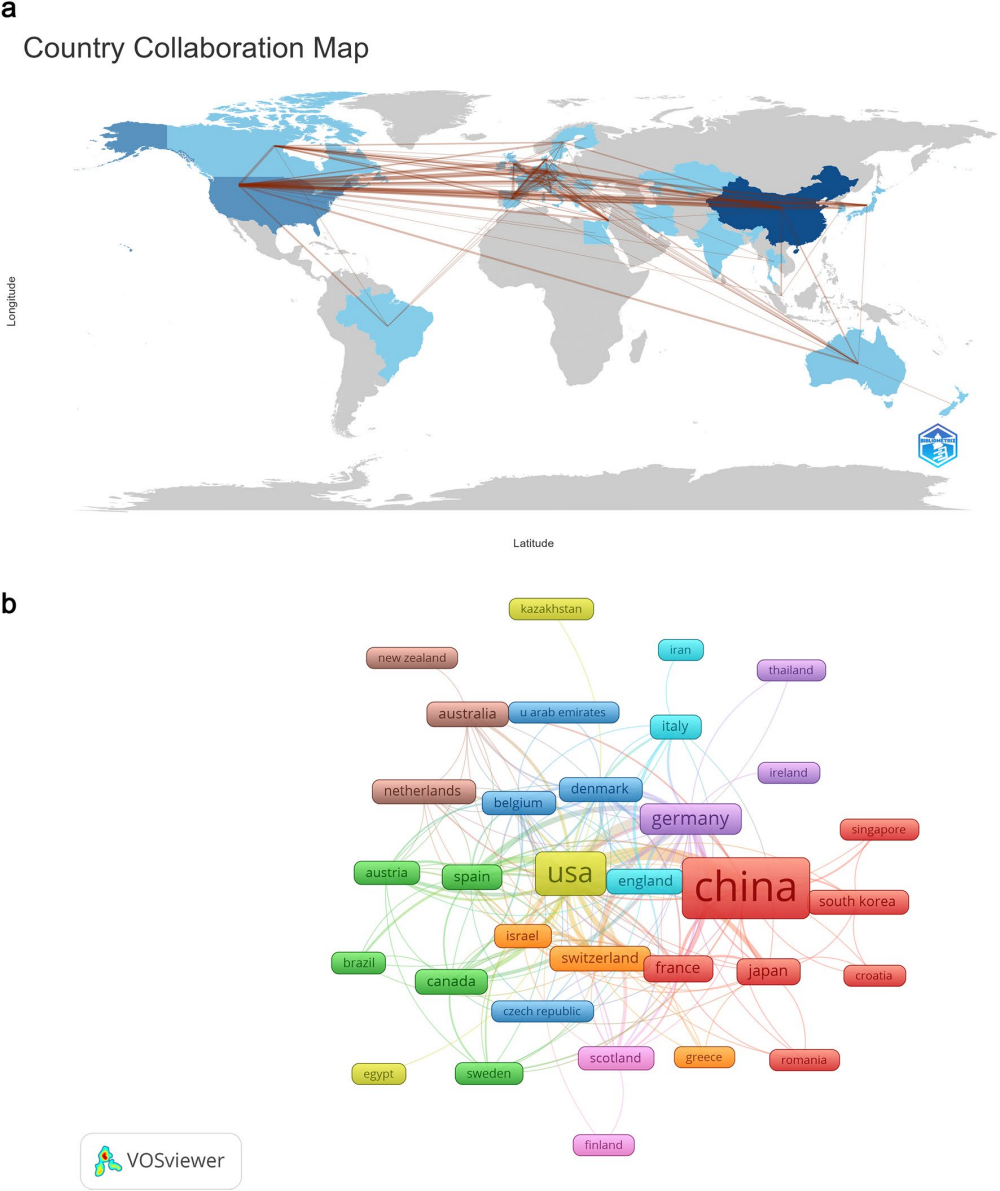
The geographical distribution、state cooperation (**A**) and co-authorship map of countries (**B**) on research of application of single cell sequencing to liver cancer

According to our search and analysis strategy, a total of 34 countries and 671 institutions were included. The top ten countries, including tied positions, are mainly distributed in Europe (*n* = 5) (Table 1). However, China has published the most articles (*n* = 210, 42.2%), followed by the United States (*n* = 95, 19.1%) and Germany (*n* = 32, 6.4%). Interestingly, the combined number of articles published by China and the United States exceeds half (61.2%) of the global number of articles published. Subsequently, we used VOSviewer to screen and visualize the 34 countries, constructing a collaboration network based on the number of publications and relationships in each country (Fig. 3). Notably, we assessed the strength of ties between countries using VOSviewer, and the results revealed significant connections between different countries. For instance, China, being the country with the highest number of articles in this field, has close ties with the United States, Germany, Denmark, and Japan. The United States, as the country with the second largest number of articles, also has strong connections with other countries, such as China, Germany, France, and Spain. However, it is worth noting that when we raise the standard for minimum tie strength, the United States becomes the center of the most dominant cooperation network, while the number of links centered on China decreases significantly. Although Japan, Denmark, and South Africa rank among the top ten countries in terms of the total number of articles published, their citation rates are not as high as those of other countries in the top ten. This suggests that the significance and depth of their articles may not be fully recognized or influential in this research field. It is noteworthy that Canada stands out with a citation-to-publication ratio of 217, surpassing other countries. The United States ranks third with a ratio of 65. In addition to publishing a larger number of articles, their high ratio of citations to documents ensures the high quality of their publications.

**Table 1:** Top 10 (including parataxis) countries and institutions on the research of application of single cell sequencing to liver cancer

Rank	Country	Counts	Institution	Counts
1	China(Asia)	210 (42.2%)	Fudan University(China)	34 (2.9%)
2	USA(North America)	95 (19.1%)	Sun Yat-Sen University(China)	19 (1.6%)
3	Germany(Europe)	32 (6.4%)	Chinese Academy of Sciences (China)	16 (1.4%)
4	France(Europe)	15 (3.0%)	Zhejiang University(China)	16 (1.4%)
5	Janpan(Asia)	14 (2.8%)	Peking University(China)	14 (1.2%)
6	Spain(Europe)	12 (2.4%)	Shanghai Jiao Tong University(China)	14 (1.2%)
7	England(Europe)	11 (2.2%)	Nanjing medical University(China)	13 (1.1%)
8	Australia(Oceania)	10 (2.0%)	University Hong Kong(China)	1 (1.0%)
9	Canada(North America)	10 (2.0%)	Central South University (China)	9 (0.8%)
10	Switzerland(Europe)	10 (2.0%)	Ministry of Education(China)	8 (0.7%)
10’			Tongji university(China)	8 (0.7%)
10’			The University of Texas MD Anderson Cancer Center (USA)	8 (0.7%)

**Fig. 4:**
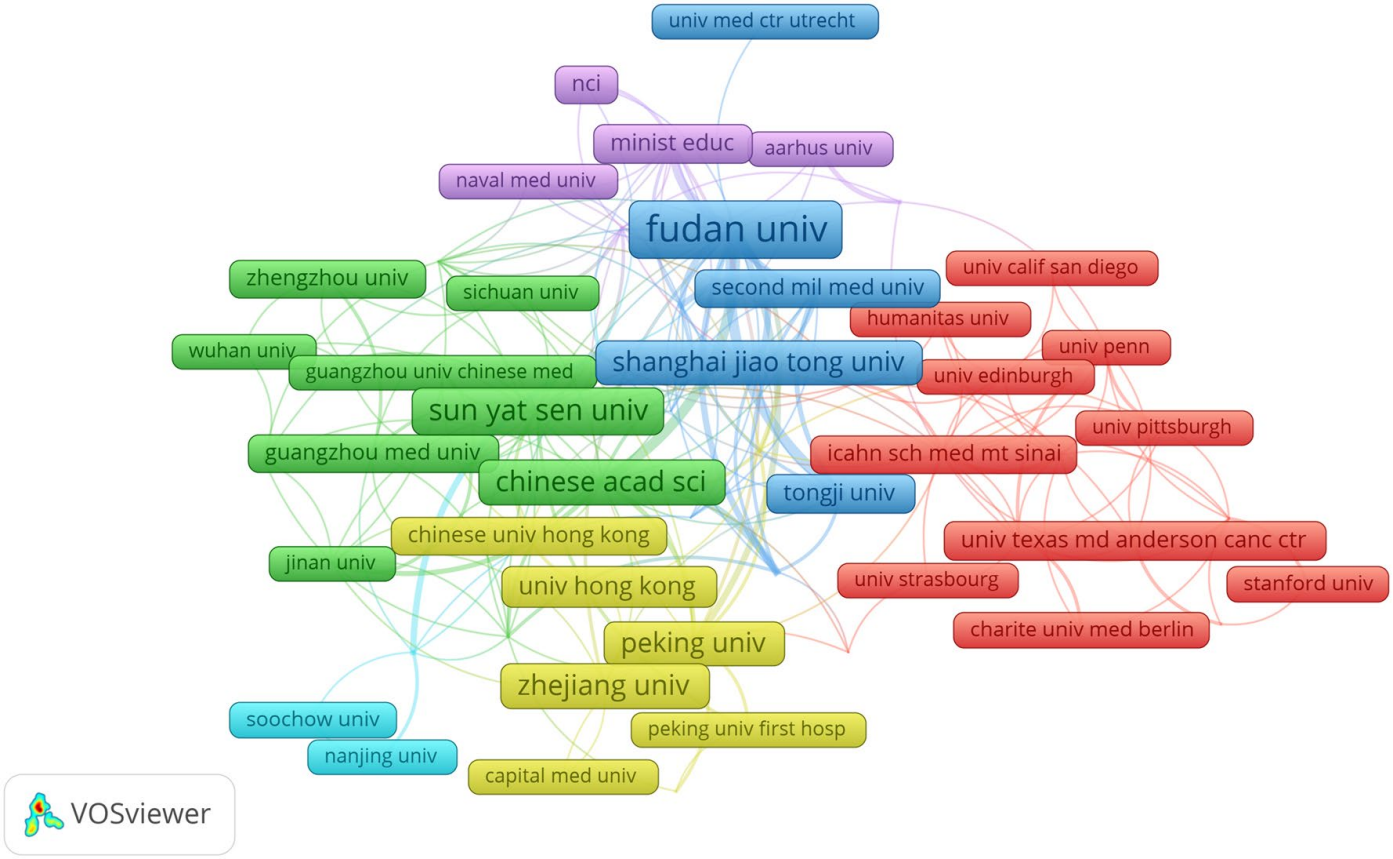
The co-authorship map of institutions on the research of application of single cell sequencing to liver cancer

After using VOSviewer to summarize, screen, and analyze the research institutions in the field, we selected the top ten institutions based on the number of publications (including parataxis), as shown in Table 1. It is noteworthy that among the top ten institutions (including parataxis) ranked by the number of publications (approximately 12 institutions), all except the University of Texas MD Anderson Cancer Center are from China. This indicates that China has devoted significant attention to the field of 'single cell sequencing applied to liver cancer'. The top ten institutions (including parataxis) collectively produced 170 articles, which accounted for 14.7% of the total output. Fudan University published the highest number of articles (Huang et al. 2019), followed by the Sun Yat-Sen University, Chinese Academy of Sciences (Pan et al. 2018), and Peking University (Pan et al. 2018). It is important to highlight that upon further analysis, we discovered that the top five institutions with the highest ratio of citations to number of publications (including parataxis) did not rank in the top ten based on the number of publications (refer to Supplementary tables in sheet 3). The first place institution, Cold Spring Harbor Laboratory, and the joint first place institution, Ontario Institute For Cancer Research, both had a ratio of citations to publications of 1810. Among the top ten institutions based on the number of publications, only the University of Texas MD Anderson Cancer Center also ranked in the top ten for the ratio of citations to number of publications. These institutions are all from the United States, indicating that articles produced by relevant institutions in the United States are of high quality. It is important for China to not only focus on the quantity of published articles but also on improving the quality and conducting in-depth research. We used VOSviewer to analyze 59 institutions that had published 4 or more articles and visualized their collaboration network in Fig. 4. The results show that Fudan University, along with the Chinese Academy of Sciences, Peking University, The Second Military Medical University of the Chinese People’s Liberation Army (not in the top ten for publication count), Tongji University, Shanghai Jiao Tong University, and others, have established a substantial and rich collaborative network. Based on our analysis, it has been observed that Zhejiang University, Sun Yat-sen University, and the University of Hong Kong have published a substantial number of papers and have engaged in collaborations with various institutions. However, upon closer examination, it appears that the level of cooperation between these universities and the collaborating institutions is relatively weak. This indicates that a strong and profound collaboration has not yet been established. In the long term, this hinders further in-depth exploration in the respective fields of these institutions. Additionally, this phenomenon may explain the relatively low ratio of citations to publications from these institutions, highlighting the need to enhance research depth and breadth by fostering a good and harmonious collaborative network.

### Journals and co-cited journals

**Fig. 5:**
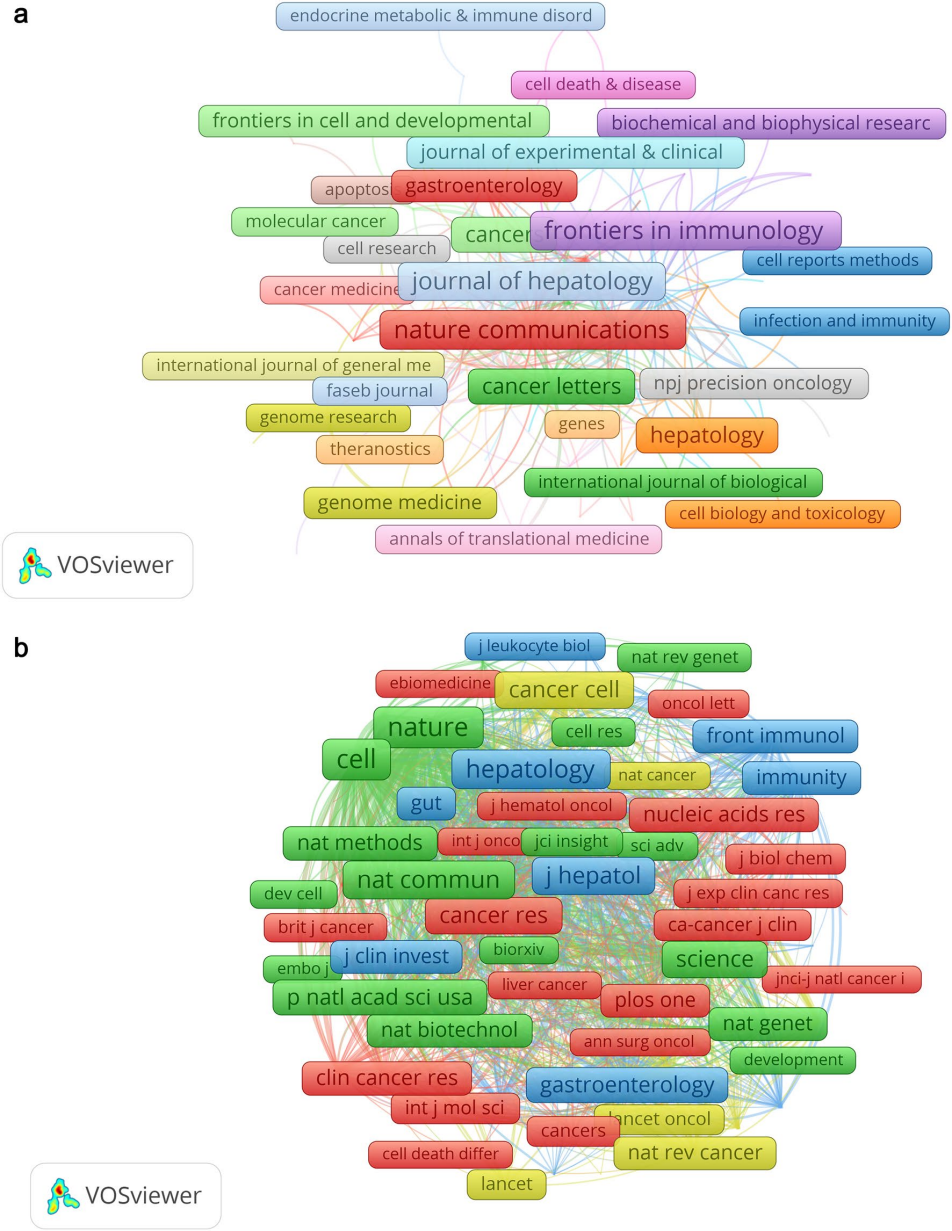
The visualization of journals (**A**) and co-cited journals (**B**) on the research of application of single cell sequencing to liver cancer

According to our search strategy, a total of 146 journals were included in this study. Among these, the journals with the most publications were ' Frontiers in Immunology' (*n* = 17, 5.1%) and ' Nature Communications' (*n* = 17, 5.1%). Among the top ten journals with the highest number of publications, 'Journal of Hepatology' had the highest impact factor (IF = 25.7), followed by 'Gut' (IF = 24.5) and 'Nature Communications' (IF = 16.6) (Table 2). Interestingly, this ranking is consistent with the ratio of citations to the number of publications (Supplementary tables in sheet 5). Additionally, among the top 10 journals with the highest number of publications, 70% of the journals are classified as Q1, which reflects the high quality of research related to the 'Application of Single Cell Sequencing in Liver Cancer' to a certain extent. To comprehensively assess the impact of these journals in the field, we utilized the bibliometrix package in R to conduct an H-index analysis. To mitigate any potential bias from solely relying on the H-index, we incorporated additional indicators such as the g-index, m-index, tc, and others in Supplementary tables in sheet 5. We then screened 56 journals based on the principle that the minimum relevant publication is equal to 2 and further drew a journal coupling analysis diagram as shown in Fig. 5A. It can be observed from the figure that journals such as 'Journal of Hepatology', 'Frontiers in Oncology', and 'Nature Communications' have extremely strong connection networks, indicating their close association with other journals. Despite being the third journal with the most published articles, Cancer Letters has not yet established a strong network of connections with other journals. As we increase the minimum value of the connection strength, the prominent role of Journal of Hepatology becomes more evident. Figure 5A also demonstrates that Journal of Hepatology serves as the core, with nodes such as 《Frontiers in Oncology》, 《Gut》, 《Nature Communications》, 《Cancers》, and 《Frontiers in Genetics》 being noticeably thicker compared to other journals. This further supports the notion that the connection between Journal of Hepatology and these journals is closer and more profound.

**Table 2:** Top 10 journals for research of application of single cell sequencing to liver cancer

Rank	Journals	Counts	2023 Impact factor	2023 JCR partition	H-index
1	Frontiers in Immunology(Switzerland)	17 (5.1%)	7.3	Q1	6
2	Nature Communications(England)	17 (5.1%)	16.6	Q1	10
3	Journal of Hepatology(Netherlands)	16 (4.8%)	25.7	Q1	11
4	Gut(England)	11 (3.3%)	24.5	Q1	7
5	Frontiers in Oncology(Switzerland)	10 (3.0%)	4.7	Q2	4
6	Cancer Letters(Netherlands)	9 (2.7%)	9.7	Q1	5
7	Cancers	9 (2.7%)	5.2	Q2	3
8	Hepatology(USA)	9 (2.7%)	14	Q1	4
9	Journal for Immunotherapy of Cancer(England)	9 (2.7%)	10.9	Q1	5
10	Frontiers in Genetics(Switzerland)	7 (2.1%)	3.7	Q2	2

After conducting a co-citation analysis using VOSviewer, we found that the top ten journals with the highest number of citations are all Q1 high-impact journals. Among them, 'Cell' ranks first with 849 citations, followed by 'Nature' with 797 citations and 'Hepatology' with 626 citations. These influential journals greatly contribute to the field of single cell sequencing in liver cancer research. We then used VOSviewer to analyze 151 journals with a co-citation number of 20 or more, constructing a co-citation relationship network shown in Fig. 5B. The network reveals that 'Cell', 'Nature', and 'Hepatology' have a strong core effect, while 'Science', 'Nature Communications', 'Gut', 'Gastroenterology', and 'Journal of Hepatology' exhibit positive co-citation relationships. This finding is further supported by increasing the minimum value of connection strength.

**Fig. 6:**
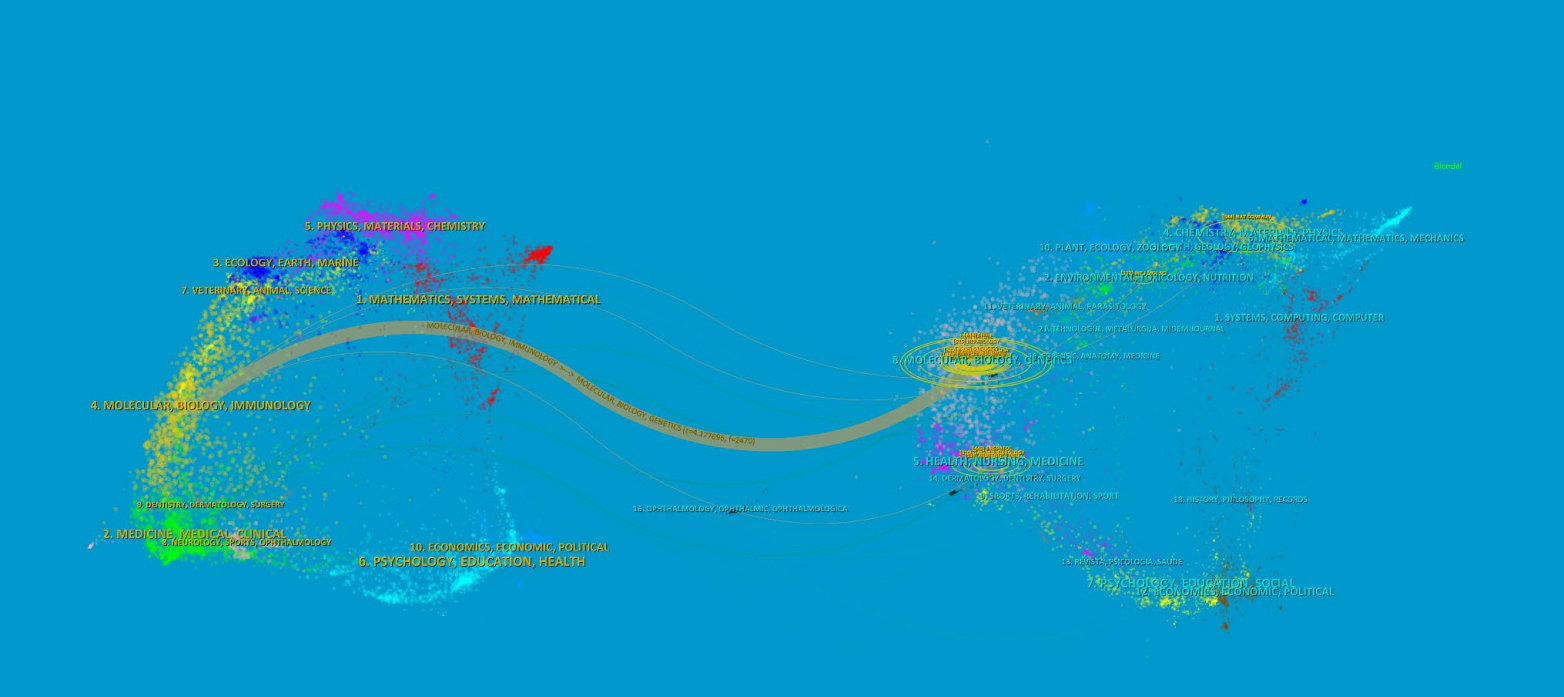
The dual-map overlay of journals on the research of application of single cell sequencing to liver cancer

To further explore the exchange of documents within a journal and the detailed interaction between multiple journals, we created a journal double-image overlay (Fig. 6). This overlay consists of a dual map that illustrates the subject distribution of an academic journal, with colored lines indicating reference paths. These lines represent the disciplines represented by the journal (Chen et al. 2023). Additionally, the double graph overlay of journals displays the citation relationship between journals and co-cited journals. The cluster of citing journals is depicted on the left, while the cluster of cited journals is shown on the right (Chen 2017). In Fig. 6, the color lines represent reference paths, with the orange line representing the main reference path (Shen et al. 2022). This indicates that articles published in Molecular/Biology/Genetics journals are primarily cited by literature in Molecular/Biology/Immunology journals (Table 3).

**Table 3:** Top 10 co-cited journals for research of application of single cell sequencing to liver cancer

Rank	Journals	Counts	2023 Impact Factor	2023 JCR partition
1	Cell	849	64.5	Q1
2	Nature	797	64.8	Q1
3	Hepatology	626	14	Q1
4	Nature Communications	544	16.6	Q1
5	Journal of Hepatology	446	25.7	Q1
6	Science	407	56.9	Q1
7	Cancer Research	387	11.2	Q1
8	Cancer Cell	358	50.3	Q1
9	Proceedings of the National Academy of Sciences of the United States of America	340	9.58	Q1
10	Gastroenterology	310	29.4	Q1

### Authors and co-cited authors

**Fig. 7:**
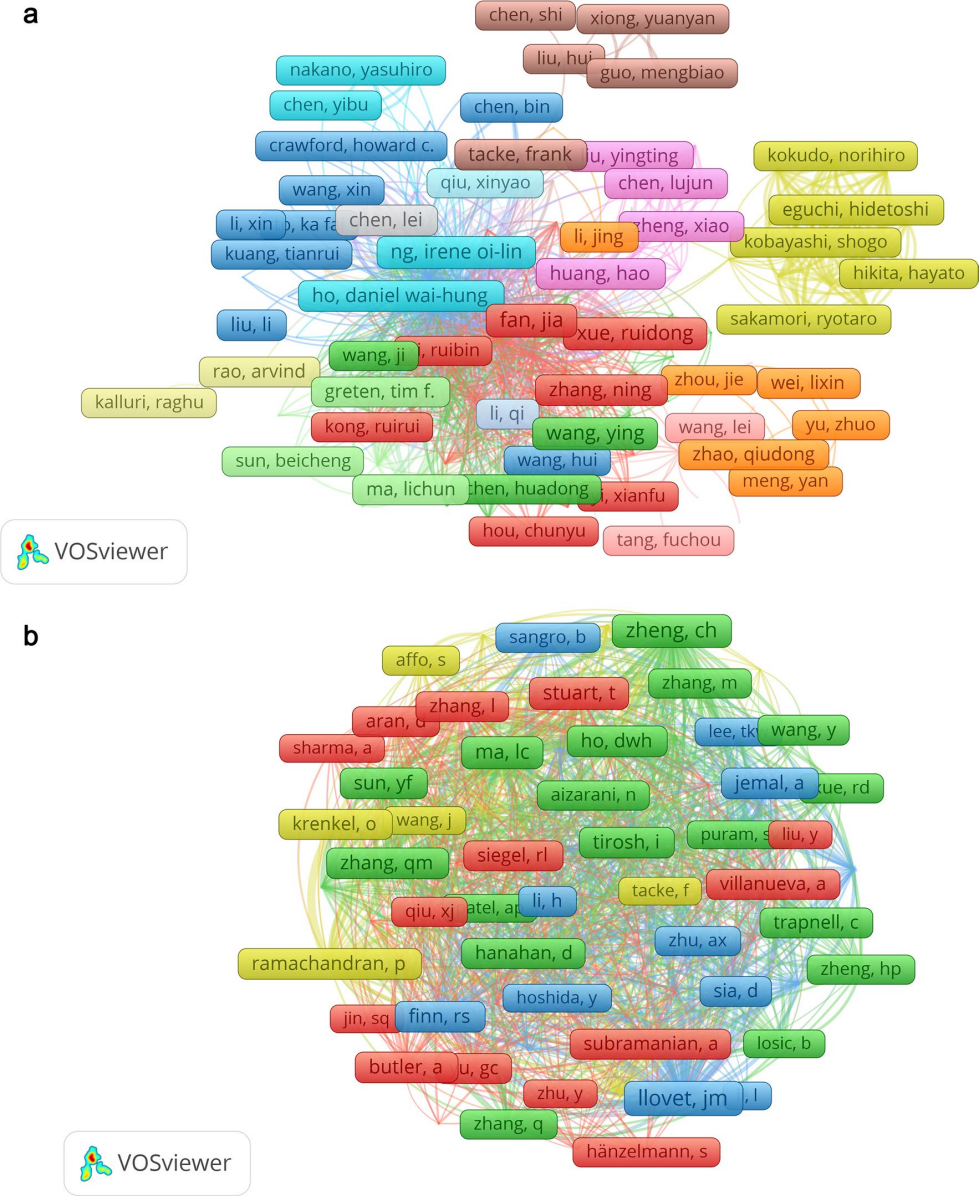
The visualization of authors (**A**) and co-cited Authors (**B**) on the research of application of single cell sequencing to liver cancer

Based on our search and analysis strategy, we included a total of 2799 authors in the field of 'Application of single-cell sequencing in liver cancer'. We ranked them according to the number of published articles and presented the top ten authors, including parataxis, in Table 4. Among these authors, Fan Jia and Zhou Jian had the highest number of publications with 5 articles each, followed by Tacke Frank, Zhang Ning, and Zhang Zemin with 4 articles each. However, it is unfortunate that none of the top ten authors in terms of publication count, including parataxis, ranked among the top ten in the 'ratio of citations to number of publications'. Among these authors, Zhang Zemin had the highest ratio of citations to number of publications (ratio of 301) (Supplementary tables in sheet 7), indicating that this author has set strict requirements for article quality while considering quantity. Additionally, we used VOSviewer to construct an author collaboration network based on the criterion that authors must have published at least 2 articles (Fig. 7A). The figure reveals that Fan Jia and Zhou Jian have the largest nodes, indicating significant collaborations. As we increase the minimum connection strength, the cooperative relationships between authors become more evident. Notably, there are strong connections between Fan Jia, Zhou Jian, Zhang Xiaoming, and Gao Qiang. The relationship between Zhang Ning and Xue Ruidong also exhibits considerable strength.

**Table 4:** Top 10 authors(Including parataxis) and Top 10 co-cited authors on research of application of single cell sequencing to liver cancer

Rank	Authors	Counts	Co-Cited Authors	Citations
1	Fan Jia	6	Llovet Jm	81
2	Zhou Jian	6	Zheng Ch	58
3	Ng Irene oi-lin	5	Ma Lc	53
4	Wang Ying	5	Stuart T	48
5	Xue Ruidong	5	Zhang Qm	43
6	Chen Lei	4	Jemal A	42
7	Ho Daniel wai-hung	4	Macparland Sa	41
8	Huang Hao	4	Sun Yf	41
9	Kuang Ming	4	Finn Rs	37
10	Liu Li	4	Macparland Sa	37
10’	Tacke Rank	4	Butler A	35
10’	Zhang Ning	4	Ho Dwh	35
10’	Zhang Zemin	4

We utilized VOSviewer to analyze co-cited authors. Out of 12,216 co-cited authors, a total of 41 authors received more than 20 citations. Llovet Jm was the most cited with 81 citations, followed by Zheng Ch with 58 citations, and Ma Lc with 53 citations. Subsequently, we selected 81 authors with a minimum of 15 citations to construct the co-cited author network presented in Fig. 7B. In the figure, the nodes representing Llovet Jm, Zheng Ch, and Ma Lc are the largest, indicating their high citation numbers. The network centered around these three authors is substantial and rich. However, as the minimum connection strength criterion increases, Zheng Ch's collaboration network becomes more prominent, suggesting a closer relationship with other authors. Notably, the connection strength between Ramachandran P and Krenkel O is much stronger than that of other authors, including Llovet Jm, Zheng Ch, and Ma Lc. This finding further confirms a closer relationship between Ramachandran P and Krenkel O compared to other authors.

### Co-Cited references and clusters

**Fig. 8:**
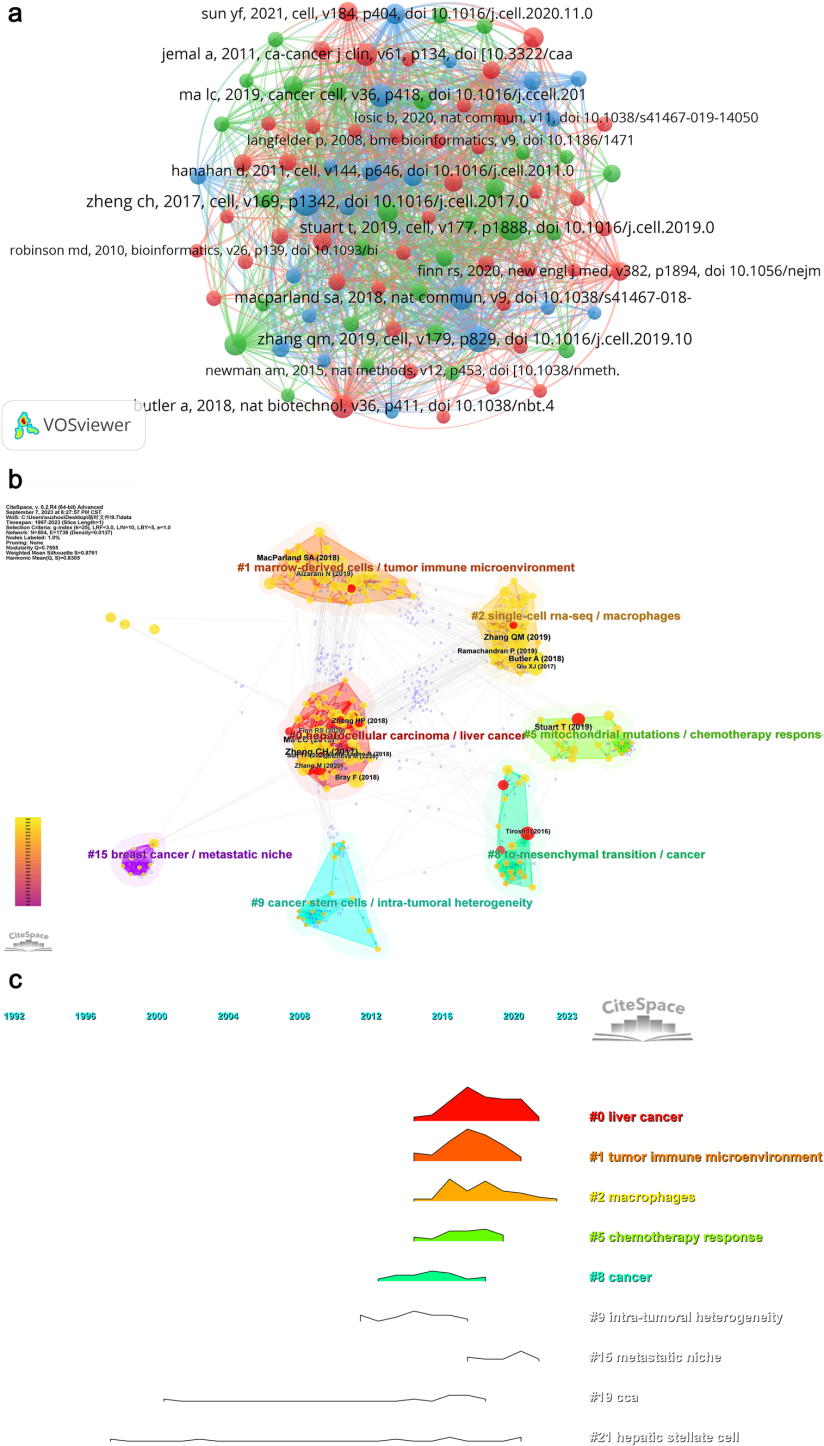
The visualization of co-cited references (**A**) 、clusters map (**B**) and Peak map(**C**) on the research of application of single cell sequencing to liver cancer

A co-citation relationship is defined as two publications being jointly cited by a third publication (Ma et al. 2021). Therefore, articles that occupy a core position in the co-citation relationship network often serve as reminders of the milestones or vane-like status in research in this field, highlighting their significance. According to our retrieval and analysis strategy, a total of 15,159 co-cited papers were included in this study. We have listed the top five citations, including parataxis, in Table 5. According to sheet 9 in the supplementary tables, it can be observed that the majority of the top 20 cited references are from Q1 journals. This ensures the feasibility and scientific rigor of the related research. Subsequently, we employed VOSviewer to construct a co-citation network for 92 papers that had a total citation count of 10 or more (Fig. 8A). As we increased the minimum co-citations threshold, we observed a positive and significant co-cited relationship network centered around the paper titled 'Landscape of Infiltrating T Cells in Liver Cancer Revealed by Single-Cell Sequencing'. This network also included papers such as 'Landscape and Dynamics of Single Immune Cells in Hepatocellular Carcinoma', 'Microenvironmental Reprogramming in Liver Cancer', 'Identification of Cancer an Immune-specific Class of Hepatocellular Carcinoma, Based on Molecular Features', 'Single-cell analysis reveals cancer stem cell heterogeneity in hepatocellular carcinoma', and 'Intratumoral heterogeneity and clonal evolution in liver cancer'. However, it is interesting to note that some articles in this network were not among the top ten cited papers (see Supplementary tables in sheet 9).

**Table 5:** Top 5 co-cited references on the research of application of single cell sequencing to liver cancer

Rank	Co-cited Reference	Doi	Citations
1	Landscape of Infiltrating T Cells in Liver Cancer Revealed by Single-Cell Sequencing (Zheng et al. 2017).	10.1016/j.cell.2017.05.035	58
2	Comprehensive Integration of Single-Cell Data.c (Stuart et al. 2019).	10.1016/j.cell.2019.05.031	46
3	Landscape and Dynamics of Single Immune Cells in Hepatocellular Carcinoma (Zhang et al. 2019).	10.1016/j.cell.2019.10.003	43
4	Tumor Cell Biodiversity Drives Microenvironmental Reprogramming in Liver Cancer. (Ma et al. 2019).	10.1016/j.ccell.2019.08.007	37
5	Single cell RNA sequencing of human liver reveals distinct intrahepatic macrophage populations (MacParland et al. 2018).	10.1038/s41467-018–06318-7	37

Afterward, we utilized CiteSpace to generate cluster analysis and peak diagrams. In Fig. 8B, we identified 7 significant clusters. The cluster number code indicates the number of keywords it contains, with smaller codes indicating more keywords (Chen et al. 2023; Li et al. 2023). Based on the analysis results, it is evident that the research on immune microenvironment, immunotherapy, and chemotherapy for liver cancer remains the focal point of this field. The peaks and ridges diagram (Fig. 8C) further supports this finding: since 2014, studies on topics such as 'tumor immune microenvironment', 'macrophages', and 'chemotherapy response' have gained prominence, surpassing the research directions of 'cca' and 'hepatic stellate cell'. Additionally, CiteSpace provides two indicators, namely modularity (Q score) and contour score (S score). The Q value ranges from 0 to 1, and a Q > 0.3 indicates a significant clustering structure. The S score represents the average cluster profile, with S > 0.5 considered reasonable and S > 0.7 indicating a highly convincing clustering result (Wu et al. 2022a), (Jin et al. 2023). In our analysis, we obtained Q = 0.79 and S = 0.88, indicating the importance of the modular structure and the effectiveness of the clustering effect.

### Reference with citation bursts

**Fig. 9:**
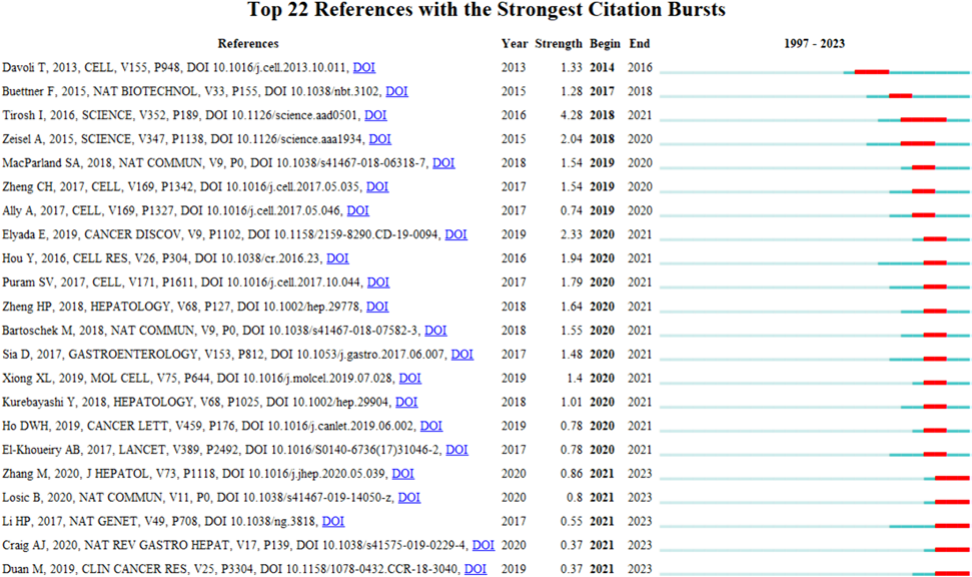
Top 22 references with strong citation bursts on the research of application of single cell sequencing to liver cancer

Citation explosive documents, also known as highly cited documents, are frequently referenced by scholars in a specific field during a certain time period (Wu et al. 2022a). In our study, CiteSpace identified a total of 22 documents with strong citation bursts (Fig. 9). The red area in the figure represents a significant citation burst (Huang et al. 2019). The figure demonstrates that the references experienced citation explosions as early as 2013 and as late as 2020. The most explosive document, titled 'Dissecting the multicellular ecosystem of metastatic melanoma by single-cell RNA-seq,' was published by Itay Tirosh et al. in the journal 'Science' with a strength of 4.28, and its citation burst occurred from 2014 to 2021. Overall, the burst intensity of these 22 documents ranges from 0.37 to 4.28, and their endurance lasts from 1 to 3 years. Notably, these documents discuss new technologies for single-cell sequencing, cellular maps of the liver, and the composition of the immune microenvironment of liver cancer. Previous studies have shown that their burst intensity is significantly higher. Table 6 provides a display and description of these explosive documents.

**Table 6:** The main research contents of the 22 references with strong citations bursts

Rank	Begin Year	Strength	Main Research Content
1	2014	1.33	The authors' team first developed the Tumor Suppressor and Oncogene (TUSON) Explorer to analyze patterns of mutational signatures in tumors and predict the likelihood of any individual gene acting as a tumor suppressor (TSG) or oncogene (OG). Further, by analyzing > 8200 tumor-normal pairs, it was concluded that "the formation of cancer genomes is through a process of cumulative single allele deficiency and triple sensitivity (Davoli et al. 2013).
2	2017	1.28	Single-cell sequencing transcriptomic methods have been developed to identify cell subsets and tease out the heterogeneity of gene expression from different sources in the single-cell transcriptome (Buettner et al. 2015).
3	2018	4.28	For the first time, the authors' team attempted to shed light on the cellular ecosystem of tumors and how single-cell genomics could shed light on targeted therapies and immunotherapies (Tirosh et al. 2016).
4	2018	2.04	Using single-cell sequencing to analyze cells in the cortex and CA1 region of the hippocampus in mice, the authors propose that transcription factors form a complex, layered regulatory code in the diversity of cortical cell types, suggesting a mechanism to maintain the characteristics of adult cell types. (Zeisel et al. 2015).
5	2019	1.54	The authors described the transcriptional profiles of parenchymal cells and non-parenchymal cells of 8444 human livers by means of single-cell RNA sequencing, fully displaying the cellular composition and characteristics of the human liver, especially the composition map of the immune microenvironment of the human liver (MacParland et al. 2018).
6	2019	1.54	The authors' team performed deep single-cell RNA sequencing on 5,063 individual T cells from peripheral blood, tumors, and adjacent normal tissues from six patients with hepatocellular carcinoma, and further determined the characteristic genes of each subset. (Zheng et al. 2017).
7	2019	0.74	The authors' team analyzed 363 cases of hepatocellular carcinoma (HCC) by whole exome sequencing and DNA copy number analysis, and 196 cases of HCC by DNA methylation, RNA, miRNA, and proteomic expression. Further, potential therapeutic targets including WNT signaling, MDM4, MET, VEGFA, MCL1, IDH1, TERT, and immune checkpoint proteins CTLA-4, PD-1, and PD-L1 were identified. (Cancer Genome Atlas Research Network 2017).
8	2020	2.33	This work identifies cancer-associated fibroblasts expressing MHC Class II, which have the ability to present antigens to CD4 + T cells and have the potential to modulate the immune response to pancreatic tumors. (Elyada et al. 2019).
9	2020	1.94	Our team provides a single-cell triomics sequencing technique, scTrio-seq, that can be used to simultaneously analyze genomic copy number variation (CNV), DNA methylation group, and transcriptome in single mammalian cells. This work lays the foundation for the analysis of the complex role of genomic and epigenome heterogeneity on transcriptome heterogeneity within cell populations. (Hou et al. 2016).
10	2020	1.79	The findings provide insight into the head and neck squamous cell carcinoma ecosystem and identify stromal interactions and epithelial-mesenchymal transformation processes associated with metastasis (Puram et al. 2017).
11	2020	1.64	The authors' team combined transcriptomic and functional analyses of hepatocellular carcinoma cells at the single-cell level to identify that different genes in different CSC subpopulations are independently associated with the prognosis of hepatocellular carcinoma, suggesting that different hepatic CSC transcriptomes influence intra tumor heterogeneity and tumor progression. (Zheng et al. 2018).
12	2020	1.55	The authors' team defined three distinct subpopulations of cancer-associated fibroblasts and revealed spatial segregation of cancer-associated fibroblast subclasses attributed to different sources, including perivascular niches, breast fat pad, and transformed epithelium. This provides the possibility for the development of biomarker driven drugs that precisely target cancer-associated fibroblasts. (Bartoschek et al. 2018).
13	2020	1.47	In an analysis of 956 patients with liver cancer, the authors found that almost 25% of liver cancer patients expressed markers of inflammatory response; Further, it was determined that some liver cancers may be susceptible to the effects of therapeutic machines designed to block T-cell regulatory pathways (Sia et al. 2017).
14	2020	1.4	The authors performed single-cell RNA sequencing on non-parenchymal cells isolated from the livers of healthy and NASH mice. Secretomic gene analysis revealed a highly connected network of disrupted intrahepatic and vascular signaling in NASH. In addition, they also identified the emergence of NASH-associated macrophages (NAMs) that are associated with disease severity and are highly sensitive to drug and dietary interventions. Finally, they indicate that hepatic stellate cells (HSCS) act as hubs of intrahepatic signaling through HSC-derived stellate factors and their response to vasoactive hormones (Xiong et al. 2019).
15	2020	1.01	The authors divided the tumor immune microenvironment of hepatocellular carcinoma into three distinct immune subpopulations, and observed varying degrees of intratumoral immune microenvironment heterogeneity, some of which reflected the multistep nature of HCC carcinogenesis, and one group identified B-cell infiltration as an independent positive prognostic factor. These results provide a basis for evaluating the immune microenvironment and general histological/molecular classification of HCC. (Kurebayashi et al. 2018).
16	2020	0.78	The authors demonstrate the feasibility and advantages of using single-cell RNA sequencing to profile intra-tumor heterogeneity and analyze the single-cell transcriptomic landscape to detect meaningful subpopulations of rare cells (Ho et al. 2019)
17	2020	0.78	The authors' team conducted clinical studies in multiple countries to evaluate the safety and efficacy of nivolumab, a programmed cell death protein-1 (PD-1) immune checkpoint inhibitor, in patients with advanced hepatocellular carcinoma with or without chronic viral hepatitis. This study demonstrated the potential of nivolumab in the treatment of advanced hepatocellular carcinoma (El-Khoueiry et al. 2017).
18	2021	0.86	The authors used single-cell transcriptome datasets to characterize intertumor heterogeneity in human intrahepatic bile duct carcinoma, highlighting the importance of intercellular crosstalk between intrahepatic bile duct cancer cells and angiocarcinoma associated fibroblasts, and revealing potential therapeutic targets (Zhang et al. 2020).
19	2021	0.8	The authors quantified intra-tumor transcriptome heterogeneity and how different components of the hepatocellular carcinoma ecosystem interact during cancer evolution. (Losic et al. 2020).
20	2011	0.55	They validated that unbiased single-cell RNA-seq analyses of tumors and matching normal samples provide a unique opportunity to characterize abnormal cell states within tumors. (Li et al. 2017).
21	2021	0.37	This paper reviews the progress of hepatocellular carcinoma and the development of related research techniques. (Craig et al. 2020).
22	2011	0.37	The authors' team revealed that the invasive mucosal associated invariant T cells of hepatocellular carcinoma function impaired and even are reprogrammed from anti-tumor immunity to pro-tumor direction. (Duan et al. 2019).

### Hotspots and frontiers analysis from keyword analysis

**Fig. 10:**
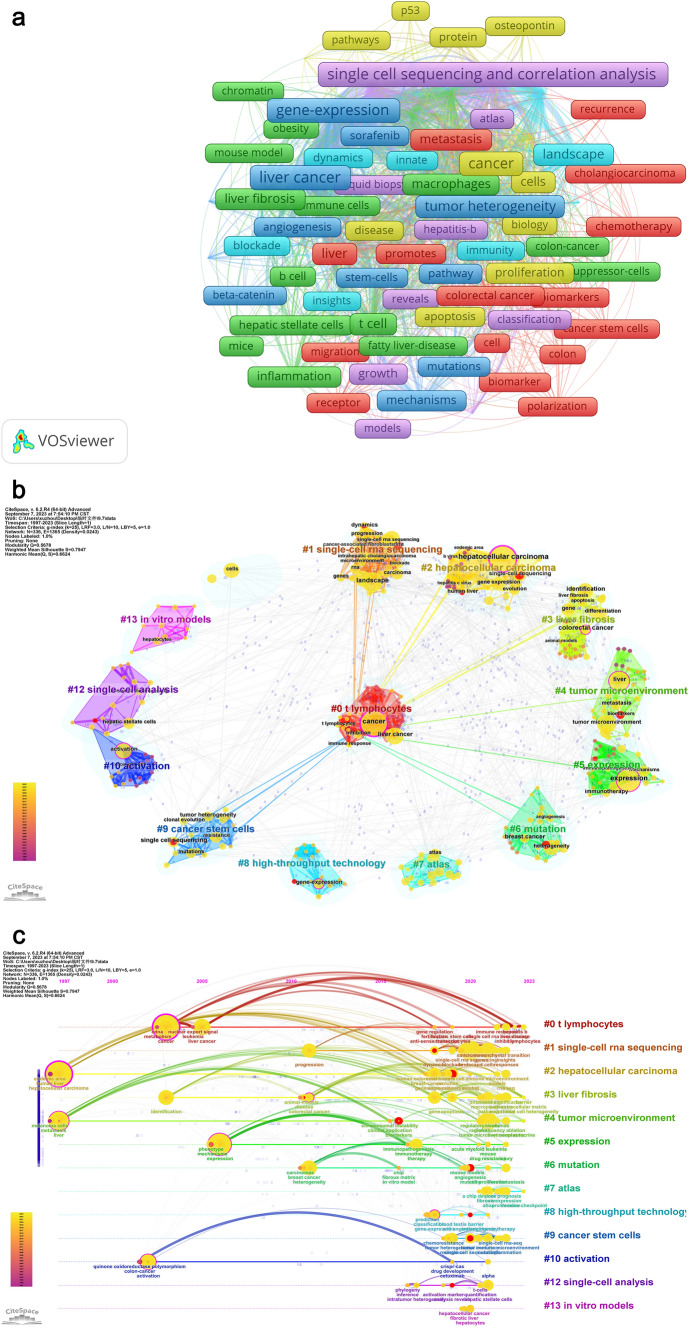

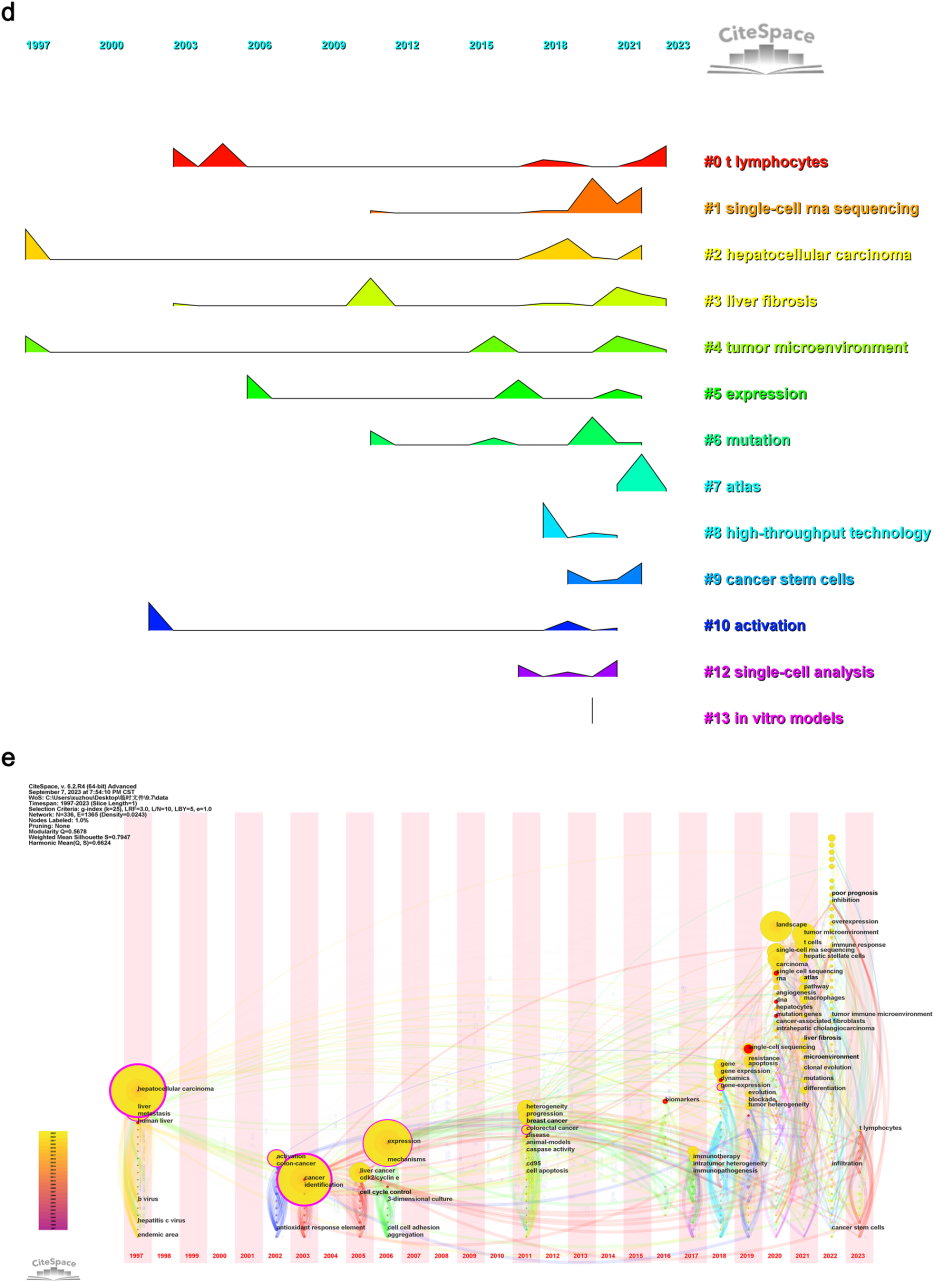
Keywords Co-occurrence analysis (**A**), cluster analysis(**B**), time flow(**C**), peak map (**D**), time zone map (**E**) on the research of application of single cell sequencing to liver cancer

In addition to the co-citation relationship network of references, the co-occurrence analysis of keywords can also provide insights into the development trends and cutting-edge research in our related fields (Wu et al. 2022a). We initially used VOSviewer to merge synonyms and eliminate invalid and blank words. Subsequently, we performed a co-occurrence analysis on the processed keywords (Fig. 10A) and identified keywords that appeared more than 30 times (Table 7). The analysis revealed that liver cancer-related gene expression, prognosis, tumor heterogeneity, and immune-related research (immune microenvironment, immune regulation, immunotherapy, T cells) form the core of liver cancer research. Single-cell sequencing technology can be utilized to delve deeper into these research areas. The results of cluster analysis further confirmed this finding (Fig. 10B). However, it is worth noting that in the co-occurrence analysis, 'liver fibrosis' and 'T cell' were not yet considered as core aspects, whereas the cluster analysis results highlight the importance of 'T cells' and 'liver fibrosis'. This suggests a potential shift in the role of 'T cells' and 'liver fibrosis' in liver cancer research, with recent studies increasingly employing single-cell sequencing to investigate their roles. Subsequently, we constructed a timeline graph (Fig. 10C) and a peak-to-peak graph (Fig. 10D) to validate our hypothesis, and the results were highly convincing (Q = 0.568, S = 0.795). To further corroborate our hypothesis and mitigate any clustering bias, additional analysis is warranted. We constructed a time zone diagram (Fig. 10E) to visualize the relationship between various research topics. In 2021, 'liver fibrosis' emerged alongside 'tumor immune microenvironment' and 'atlas'. It is evident that subsequent studies will delve deeper into 'liver fibrosis' by exploring its transformation into fibroblasts, which can be attributed to advancements in single-cell sequencing technology. Similarly, 'T cells' have been extensively studied and are still a primary focus of research. Notably, studies on the tumor immune microenvironment and tumor-related gene expression span the entire timeline, indicating their significance in both co-occurrence and cluster analysis.

**Table 7:** Keywords that appear more than 30 times on research of application of single cell sequencing to liver cancer

Rank	Keywords	Counts	Rank	Keywords	Counts
1	Liver cancer	158	8	Tumor Heterogeneity	54
2	Single cell sequencing and correlation analysis	137	9	Immunotherapy	53
3	Gene-Expression	130	10	T Cell	45
4	Cancer	115	11	Landscape	42
5	Prognosis	92	12	Liver	39
6	Tumor immune microenvironment	56	13	Cells	32
7	Immunoregulation	54	14	Macrophages	31

## Discussion

### General information

The analysis of published literature, including statistics, summaries, and visual analysis, can provide valuable insights into the research status and hot topics in a particular field (Wilson et al. 2021). In this study, we included a total of 331 published documents from 34 countries, involving 671 institutions and 2799 original authors. Based on an analysis of annual publication volume, it has been observed that the publication volume has shown close to exponential growth since 2020. This can be attributed to the continuous improvement and standardization of single-cell sequencing technology, as well as the researchers who have been assisted by this technology. These advancements have enabled researchers to accurately depict the cellular map of the liver, explore immune regulation and potential treatments for liver cancer, and construct the immune microenvironment of liver tumors with precision. In order to explore this field further, a comprehensive analysis of co-cited documents and explosive references plays a crucial role.

A co-citation relationship is defined as the joint citation of two publications by a third publication. This relationship reflects the research focus in a specific field (Chen et al. 2023; Ma et al. 2021). Explosive citations indicate a peak in research activity (Wu et al. 2022a). In addition to a report that maps tumors and describes the current global cancer burden (Sung et al. 2021), the Phase II articles primarily focus on the perspectives of 'tumor immune microenvironment', 'tumor heterogeneity', 'immune regulation and immunotherapy', and 'single-cell technology update'. Regarding the research direction of tumor immune microenvironment, researchers conducted deep single-cell sequencing and transcriptomic analysis on T cells. They then performed rigorous clustering based on functional and molecular characteristics. The developmental trajectory of T cells (activation, clonal expansion, depletion, etc.), intercellular interactions, characteristic genes, and potential targets were comprehensively described (Zheng et al. 2017; Tirosh et al. 2016). This process involved the exploration of liver cancer specimens and supplementary analysis of other tumor specimens.In order to comprehensively understand the cellular composition of the liver, the researchers performed single-cell sequencing and transcriptomic analysis on fresh human liver samples. This analysis provided a detailed transcriptional profile of various cell types including parenchymal cells, lung parenchymal cells, liver cells, endothelial cells, bile duct cells, liver stellate cells, B cells, conventional and unconventional T cells, and other immune cells (MacParland et al. 2018). Additionally, the researchers investigated the relationship between tumor-associated macrophages and poor prognosis, the dynamic characteristics of CD45 + cell types, and the origin of myeloid and lymphoid cells in ascites, which expanded our knowledge of the tumor immune microenvironment (Zhang et al. 2019). Furthermore, this analysis also included the initial studies that described the six functions of tumor growth and development, which led to the concept of tumor immune microenvironment (Hanahan and Weinberg 2011). Immune regulation and immunotherapy play a crucial role in liver cancer research. By focusing on liver cancer, researchers have discovered a negative correlation between p53 and survival, as well as identified potential immunotherapeutic targets such as WNT signaling, MDM4, MET, VEGFA, MCL1, IDH1, TERT, and immune checkpoint proteins CTLA-4, PD-1, and PD-L1 (Cancer Genome Atlas Research Network 2017). The single-cell sequencing analysis of liver cirrhosis and fibrosis not only identified and defined specific subsets of macrophages and endothelial cells, but also laid the foundation for studying human organ fibrosis from an immune and molecular perspective (Ramachandran et al. 2019). The heterogeneity of tumor cells is a crucial factor contributing to treatment failure and fatal outcomes in solid malignancies. In order to illustrate the transcriptional heterogeneity of cell cycle, space, and drug resistance programs exhibited in malignant cells, the researchers conducted a comprehensive investigation of liver cancer and supplemented it with single-cell sequencing analysis of other cancers (Tirosh et al. 2016). They also observed varying degrees of heterogeneity in the tumor immune microenvironment (Ma et al. 2019). Although single-cell sequencing and its related analysis have made significant progress, there are still some gaps and complementary links in the second phase of publication. As a result, researchers at this stage have developed different algorithms or models for exploratory innovation. It is worth noting that these models continue to provide valuable insights and may guide the development of new algorithms or models. The first such model is gene enrichment analysis (Subramanian et al. 2005). Although this concept has been thoroughly analyzed and applied, the interaction and complementarity between gene enrichment analysis and single-cell sequencing remain important analytical tools that should not be overlooked. Subsequently, patterns of mutational features in tumors can be analyzed, and the likelihood of any individual gene acting as a tumor suppressor or oncogene can be predicted. The emergence of tumor suppressor and oncogene (TUSON) explorer models has provided a foundation for explaining recurrence patterns of aneuploidy in cancer (Davoli et al. 2013). Subsequently, the proposed single-cell latent variable model has allowed researchers using single-cell sequencing technology to exclude the influence of potential confounders, such as cell cycle, on gene expression heterogeneity. This model has also provided a possible method to identify the different sources of gene expression heterogeneity in the single-cell transcriptome (Buettner et al. 2015). The introduction and improvement of shared population and downstream comparative analysis across data sets have further supported the identification of cell subpopulations existing in multiple data sets. This advancement has deepened our understanding of how different cell states respond to perturbations, diseases, and evolution (Stuart et al. 2019), (Butler et al. 2018).

Of the 34 countries in our study, China and the United States lead in terms of research volume, with their combined publication volume exceeding that of the other 31 countries. The main risk factors for liver cancer include chronic hepatitis B virus or hepatitis C virus infection, aflatoxin-contaminated foods, heavy alcohol consumption, excess weight, type 2 diabetes, and smoking (Sung et al. 2021; Thun et al. 2017). Unfortunately, China previously had a high density of people exposed to hepatitis B virus and aflatoxin, indicating a significant potential for liver cancer (Li et al. 2013), (Rahman et al. 2015), (Ma 2018), (Yan et al. 2022), (Chimed et al. 2017). This evidence supports the substantial effort invested by Chinese researchers in liver cancer research. The application of single-cell sequencing in liver cancer research requires substantial investment and high-tech support to ensure accurate results and analysis. Developed countries like the United States and Germany are well-equipped to meet these requirements, which may explain their dominant position in this field and their ability to form larger and more interconnected networks compared to China. The publication volume ranking of an institution is often consistent with the publication volume ranking of a country. When analyzing the topic of single-cell sequencing applied to liver cancer research, we found that only one of the top ten institutions in terms of publication volume is from the United States (University of Texas MD Anderson Cancer Center), while the other institutions are from China. Unfortunately, only the University of Texas MD Anderson Cancer Center ranks among the top ten institutions with the highest number of publications in the 'ratio of citations to publications' ranking, which reflects the quality of scientific research. This can be attributed to the fact that single cell sequencing requires significant financial investment and advanced technological support to ensure accurate results and analysis. Additionally, it is observed that many excellent institutions or universities in China have not yet established a comprehensive network of partnerships. In the long term, establishing strong cooperative relations between institutions and promoting international collaboration can enhance the depth and breadth of research in this field. This comprehensive improvement and widespread adoption of single-cell sequencing technology can greatly contribute to liver cancer research, leading to a better understanding of tumors and laying the foundation for precise treatment and effective control.

The application of single-cell sequencing to liver cancer has been extensively studied and published in reputable journals such as Nature Communications and the Journal of Hepatology. Through our integration and visual analysis, we discovered that 60% of the top 10 journals, in terms of number of publications, are categorized as JCR Q1 journals. This finding further supports the notion that the research conducted in this field is of high quality and significance. Moreover, our investigation revealed that the top ten cited journals are also classified as Q1 journals. Notably, the three major journals recognized in the field, namely Cell, Nature, and Science, have made exceptional contributions. This observation reinforces the reliability of the theoretical background and the recognition of relevant research techniques. In addition to the comprehensive journals in the top ten, the other journals that made significant contributions are evenly distributed in specialized journals focused on 'immunity' and 'heredity' (or genes). This finding aligns perfectly with the results of our double-graph overlay analysis. However, after Z conversion, the term 'Clinical' also applies to various fields such as 'Genetics' and 'Medicine', although its application intensity is relatively weak. This suggests that further exploration is needed to guide liver composition and the immune microenvironment of liver cancer through single-cell sequencing analysis, to improve clinical diagnosis, treatment, and prognosis. It is also important to note that Asia and Africa have the highest incidence of liver cancer cases. Despite extensive research on risk factors such as alcohol consumption, HBV virus, and aflatoxin virus, diseases like primary liver cancer or cholangiocarcinoma continue to pose significant health threats, particularly in countries undergoing transition in Asia and Africa (Sung et al. 2021), (Prueksapanich et al. 2018), (Petrick et al. 2017, 2020), (Welzel et al. 2007), (Donato et al. 2001), (Arnold et al. 2020). In contrast, the main published and co-cited journals are predominantly from Europe and North America, highlighting the need for the establishment and development of scientific research journals in Asia and Africa.

Fan Jia and Zhou Jian, both affiliated with Zhongshan Hospital and Fudan University, have made significant contributions to the application of single-cell sequencing technology in liver cancer research. Our analysis reveals a strong collaboration between the two teams. In our study, we have combined their research findings. Throughout their research, Fan Jia, Zhou Jian, and their team have successfully applied single-cell sequencing technology to various aspects of liver cancer. Their primary focus has been on cholangiocarcinoma, where they have utilized global sequencing, single-cell sequencing, and single-sample genome analysis. These techniques have allowed them to identify the genomic heterogeneity within the immune infiltrating state of intrahepatic cholangiocarcinoma. Additionally, they have explored the immune infiltrating environment, identified prognostic markers, and provided detailed classifications and descriptions of T cells and B cells, relating them to the prognostic state. Their research not only enhances our understanding of the immune infiltrating state and immune microenvironment of intrahepatic cholangiocarcinoma but also offers new insights for immunotargeted therapy (Chen et al. 2021; Lin et al. 2022; Song et al. 2022). In addition to studying cholangiocarcinoma, the researchers also investigated and analyzed peripheral circulating tumor cells. They proposed the exploration of spatial heterogeneity and described the immune escape mechanism of circulating tumor cells (Sun et al. 2021a). Additionally, they developed the ChimeraX-i120 platform, which enables negative enrichment of circulating tumor cells, immunofluorescent labeling, and machine learning-based recognition. They emphasized that this platform is effective in clinical applications (Wang et al. 2021). Apart from the ChimeraX-i120 platform, they also pioneered the development of a computational pipeline called scMetabolism for quantifying single-cell metabolism. This pipeline was used to observe the activity of macrophages, laying a foundation for exploring the heterogeneity of liver metastasis in colorectal cancer, and the immune microenvironment, and evaluating the effect of neoadjuvant chemotherapy (Wu et al. 2022b). Furthermore, their team conducted innovative research using single-cell sequencing technology to study the evolution mechanism of different tumors in liver cancer (Duan et al. 2018).

### Review with hotspots and frontiers from keywords analysis

In addition to analyzing co-cited literature and explosive citations, keyword analysis can provide insights into knowledge distribution, correlation, and future research directions in related fields (Wang et al. 2022). Our analysis reveals that the research directions of 'tumor heterogeneity', 'tumor immune microenvironment (including T cells and macrophages)', 'immune regulation and therapy', and 'prognosis and outcome of liver tumors' hold a central position in both literature and keyword analysis. These areas of research have demonstrated significant potential and momentum in recent years. Cluster analysis, peak maps, and even time maps and time zone maps further support this viewpoint. Therefore, we have consolidated the aforementioned research directions and present them as follows:

## Liver tumor heterogeneity

Liver tumors pose a significant challenge in global public health due to their heterogeneity and complex pathophysiological mechanisms. This heterogeneity is evident in various aspects including gene mutations, gene expression patterns, epigenetic changes, metabolic differences, and immune response (Moeini et al. 2016). Genetic heterogeneity is a key characteristic of liver tumors, reflecting the diverse gene mutations and expression patterns present in these tumors (Zucman-Rossi et al. 2015). This complexity contributes to the resistance of liver tumors to treatment (Wang et al. 2012). Epigenetic heterogeneity also plays a crucial role in the development and progression of liver tumors, involving DNA methylation, histone modification, and non-coding RNA changes. These epigenetic alterations significantly impact the progression of liver tumors (He 2020). Metabolic heterogeneity encompasses differences in metabolic profiles within different regions of the tumor and metabolic interactions between tumor cells and their microenvironment. These metabolic differences not only influence the biological behavior of liver tumors but also affect their response to therapy and prognosis. The heterogeneity of immune response in liver tumors results in variations in the effectiveness of immunotherapy. This includes the interaction between tumor cells and immune cells, as well as the formation of immunosuppressive microenvironments (Ringelhan et al. 2018), (Huang et al. 2021). Immune response heterogeneity, also referred to as tumor heterogeneity, can be classified into tumor spatial heterogeneity and temporal heterogeneity, thanks to advancements in single-cell sequencing technology.

The occurrence and progression of tumors are influenced not only by the genetic variation of tumor cells themselves but also by the microenvironment that supports tumor growth. Recent research has revealed significant heterogeneity in the characteristics of the microenvironment at different locations within the tumor. This spatial heterogeneity plays a crucial role in regulating the biological behavior of the tumor (Xue et al. 2022). The tumor microenvironment exhibits spatial heterogeneity in terms of nutrition and oxygenation conditions, pH value, cytokine distribution, cell composition, functional status, and extracellular matrix composition. The central region of the tumor experiences hypoxia and acidosis due to limited tumor angiogenesis and poor blood vessel permeability, while conditions are relatively better in the marginal region. The concentrations of cytokines and growth-promoting factors secreted by various tumor-related cells also exhibit a gradient distribution within tumors. The proportions of immune cells, tumor cells, and stromal cells within the tumor can vary (Zheng et al. 2020). Furthermore, there are differences in cell phenotypes and genetic characteristics between the tumor center and the invasive edge (Wu et al. 2021).

Several studies have demonstrated that the spatial heterogeneity of the tumor microenvironment can impact various aspects of tumor biology, such as tumor growth rate, invasion, metastasis ability, and therapeutic responsiveness. In a study investigating the spatial heterogeneity of liver cancer circulating tumor cells (CTCs) using single-cell RNA sequencing, Sun et al. identified chemokine CCL5 as a crucial mediator of immune escape in hepatic CTCs. They observed that overexpression of CCL5 is regulated by transcription of p38-MAX signal transduction, which recruits regulatory T cells (Treg) to promote immune escape and metastatic spreading of hepatic CTCs. Interestingly, this phenomenon is rarely observed in hepatic in situ tumors (Sun et al. 2021a). Therefore, conducting in-depth analyses of the spatial heterogeneity of the tumor microenvironment holds immense significance in the development of novel therapeutic strategies targeting the tumor microenvironment. Currently, the utilization of various omics techniques to explore tumor heterogeneity has emerged as a prominent research area, offering the potential to unveil new mechanisms underlying tumor progression.

With the in-depth study of tumor microenvironment, researchers have increasingly recognized that tumor microenvironment exhibits not only spatial heterogeneity but also significant temporal heterogeneity during tumor development. The dynamic changes in temporal heterogeneity have a crucial impact on tumor progression (Losic et al. 2020). In the early stages of tumor development, the immune microenvironment plays a role in inhibiting tumor growth. However, as cancer progresses, tumor-related inflammatory factors and immunosuppressive cells accumulate, leading to a weakened anti-tumor immune response. Moreover, the formation of tumor blood vessels is a dynamic process, where the formation of new blood vessels stimulates tumor proliferation. Furthermore, as treatment progresses, both tumor cells and the microenvironment undergo various changes in response (Dong et al. 2018). For instance, chemoradiotherapy can induce immune cell aggregation and vascular regeneration (Liu et al. 2023). Unveiling the temporal heterogeneity of the tumor microenvironment would facilitate better monitoring of tumor progression and the development of corresponding treatment strategies. The rapid advancement of single-cell techniques has opened up new avenues for studying the heterogeneity of liver tumors. Single-cell transcriptomics enables the analysis of individual cells within liver tumor tissues, thereby revealing differences in the expression patterns of cell subsets in different regions. This approach provides researchers with a more detailed understanding of the distribution and functional status of different cell types within liver tumors, ultimately serving as a more accurate basis for personalized treatment. By analyzing the heterogeneity of liver tumors, it is evident that tumor cells in different regions exhibit varying proliferation ability, metastasis potential, and drug resistance. Furthermore, the blood vessel supply and distribution of immune cells within the tumor are closely associated with tumor development and response to treatment. Consequently, the development of personalized treatment strategies for liver tumors in different regions holds great promise in enhancing treatment efficacy and survival rates.

## Liver tumor immune microenvironment

In recent years, cancer immunotherapy has gained significant attention, particularly immune checkpoint inhibitor therapy. This type of drug has shown promise in effectively identifying and attacking tumor cells by targeting inhibitory signaling pathways on immune cells. As a result, cancer patients can potentially benefit from improved therapeutic outcomes. However, it has been observed in clinical practice that only a small subset of cancers respond well to this therapy, while a significant number of tumors are not sensitive to immune checkpoint inhibitors. Liver tumors, in particular, exhibit high immune heterogeneity, and their development and progression are closely associated with the immune microenvironment. This microenvironment consists of various components, including tumor cells, immune cells, cytokines, and suppressor cells (Donne and Lujambio 2023). Liver tumor cells can evade immune responses by manipulating antigen presentation pathways, thereby inhibiting the activation and function of immune cells. Immune cells present in the liver tumor microenvironment, such as T cells, natural killer cells (NK cells), dendritic cells (DCs), and tumor-associated macrophages (TAMs), play a crucial role in tumor development and prognosis. Additionally, cytokines like tumor necrosis factor (TNF), interferon (IFN), and interleukin (IL) found in the immune microenvironment of liver tumors are involved in regulating the activation, proliferation, and function of immune cells. Furthermore, suppressor cells, including regulatory T cells (Tregs) and myeloid suppressor cells (MDSCs), contribute to immune escape by inhibiting the activation and function of immune cells within the liver tumor microenvironment. The immune microenvironment of liver tumors is not only complex in composition but also involves intricate interactions among various components. Liu et al. conducted a study using ScRNA-seq to construct an immune microenvironment map of human healthy liver tissue, adjacent cancerous tissue, and tumor tissue. They discovered that the dysregulation of the immune microenvironment in liver tumors is caused by the cross-talk mechanisms of SPP1-CD44, IF-TNFRSF14, and VEGFA-NRP1 (Liu et al. 2022a). In a similar approach, Ma and Liu et al. found that LGALS9-SLC1A5, SPP1-PTGER4, and SPP1-CD44 tumor cells interact with macrophage receptor-ligand pairs, which are associated with tumor aggressiveness (Ma et al. 2022; Liu et al. 2022b). Tumor cells release chemical factors and cytokines to regulate the function and number of immune cells, while immune cells secrete cytokines and mediate intercellular signal transduction to affect the proliferation, apoptosis, and invasion of tumor cells. For instance, YAP signaling activates tumor-associated fibroblasts (CAF), leading to the interaction between COL1A1-ITGA2 and tumor cells, which mediates transcriptional diversity in liver tumor cells (Meng et al. 2022). Suppressor cells inhibit the activation and function of immune cells, thereby weakening the tumor immune response (Giraud et al. 2021). Single cell sequencing techniques have revealed that tumor-associated macrophages inhibit tumor T cell infiltration in the immune microenvironment of HBV-associated hepatocellular carcinoma. The interaction between TIGIT and NECTIN2 regulates the immunosuppressive environment, transforming immune cells into a more immunosuppressive state and reflecting an overall cancer-promoting immune cell landscape (Ho et al. 2021).

### Liver tumor-infiltrating T cells

Single-cell sequencing technology enables the examination of gene expression and epigenetic changes at the individual cell level, offering a novel approach to investigating the heterogeneity of T cells within the liver tumor microenvironment. Recent studies have utilized single-cell RNA sequencing to analyze T cell subsets in both tumor tissue and peripheral blood of liver cancer patients. These studies have revealed that T cells in tumor tissue exhibit functional depletion and increased expression of multiple inhibitory receptors. Additionally, other studies have employed single-cell whole genome sequencing to characterize the clonal diversity of CD8 + T cells and the composition of the TCR complex at the site of liver cancer lesions. These findings contribute to our understanding of the mechanisms through which the tumor microenvironment inhibits T cell function and provide a theoretical foundation for the development of liver cancer immunotherapy. However, further investigations are needed to expand the sample size and integrate multiple single-cell omics techniques to describe the dynamic changes in T cell heterogeneity during the progression of liver cancer.

Single-cell sequencing technology enables accurate identification of immune cell types present in the tumor microenvironment. In two separate cohorts of primary hepatocellular carcinoma and recurrent hepatocellular carcinoma, there was a decrease in the number of Treg cells in the recurrent hepatocellular carcinoma. Conversely, dendritic cells (DC) and infiltrating CD8 + T cells increased and exhibited overexpression of KLRB1, while demonstrating low cytotoxicity and clonal expansion. This is in stark contrast to the mostly depleted state observed in primary liver cancer (Sun et al. 2021b). The use of single-cell technology allows for simultaneous analysis of tumor and immune cells. A study utilizing single-cell sequencing analyzed liver cancer cells and immune cells in tumor tissues of liver cancer patients and discovered a correlation between CD8 + T cells and the ratio of M2 macrophages, revealing potential mutual regulation between them (Ho et al. 2021). Additionally, the dynamic alteration of the tumor immune microenvironment was observed through the interaction between SLC40A1, GPNMB macrophage populations, LAMP3 dendritic cell populations, and infiltrating T cells (Zhang et al. 2019). Therefore, single-cell technology not only facilitates the study of interactions between tumor cells and immune cells, but also enables immune detection and prognosis assessment of liver tumors in neighboring normal tissues, peripheral blood, and distant lymph nodes.

Through a comprehensive analysis of recent literature, it has been determined that the future application of single-cell technology in studying the immune microenvironment of liver tumors can be categorized into the following main directions:1. Monitoring the dynamic response to immunotherapy: Utilizing single-cell sequencing technology, the changes in the composition and function of T cells in tumor tissues and peripheral blood of patients before and after immunotherapy, such as PD-1 inhibitor treatment, can be monitored. This evaluation of treatment response aims to identify potential drug resistance mechanisms.2. Humoral immune monitoring: By detecting circulating tumor-reactive T cells, it is possible to achieve non-invasive monitoring of the tumor immune environment and optimize treatment plans.3. Multi-omics data integration: The integration of single-cell transcriptome, TCR sequencing, and epigenetic data allows for an in-depth analysis of individual T cell functions. This analysis can provide precise guidance for individualized immunotherapy.4. Customized targeted therapy: By identifying potential tumor antigen peptides and their reactive TCR, it becomes possible to design precision immunotherapy strategies, such as novel TCR-engineered CAR-T. This technique proves particularly important in exploring the process of tumor development.

### Liver tumor macrophages

Through recent literature searches on single-cell sequencing technology in liver tumor immune microenvironment, it has been observed that tumor-associated macrophages (TAM) have been extensively studied. Multi-dimensional analysis has confirmed that TAMs are among the most abundant immune cells infiltrating the liver tumor microenvironment (Cheng et al. 2022). TAMs in liver tumors have various functions, including anti-tumor immune surveillance, antigen presentation, cytotoxicity, and regulation of the tumor microenvironment. By recognizing and phagocytosing tumor cells, macrophages promote antigen presentation and T cell activation, thereby enhancing tumor immune surveillance. Furthermore, macrophages produce a range of cytokines and chemicals that directly impact tumor cells, while also regulating inflammatory responses and angiogenesis in the tumor microenvironment. ScRNA-seq data demonstrate that specific TAMs play a central role in tumorigenesis and therapeutic resistance through interactions with various cell populations in the HCC tumor microenvironment (Cheng et al. 2022), (Sung 2022).

The polarization state of liver tumor-associated macrophages plays a crucial role in their anti-tumor effect. Macrophage polarization refers to their functional characteristics in the tumor microenvironment and can be categorized into two states: M1 type and M2 type macrophages. M1 macrophages are activated and have anti-tumor and anti-inflammatory effects. Activation of M1 macrophages leads to the release of various anti-tumor cytokines and chemicals, including tumor necrosis factor-α (TNF-α), interferon-γ (IFN-γ), nitric oxide (NO), and interleukin-12 (IL-12), among others. These molecules are capable of directly killing tumor cells, promoting antigen presentation, T cell activation, and enhancing the local immune response. On the other hand, M2 macrophages are in an immunomodulatory state that primarily regulates the tumor microenvironment through anti-inflammatory and immunosuppressive effects. Cytokines produced by M2-type macrophages, such as interleukin-10 (IL-10), transforming growth factor-β (TGF-β), and anti-inflammatory cytokine α (IL-1Ra), can inhibit immune cell activation and immune-mediated killing of tumor cells, while promoting angiogenesis and tumor growth (Cheng et al. 2022). The activation of M1-type macrophages can enhance the anti-tumor immune response by directly killing tumor cells and promoting the activation of T cells. On the other hand, an increase in M2-type macrophages may lead to immunosuppression and tumor escape. Therefore, regulating the polarization of macrophages is an important strategy for the treatment of liver tumors. For instance, a recent study discovered that the expression of APOC1 in TAM of HCC tissue was higher compared to normal tissue. Through liver tumor tissue, paracancer tissue, and peripheral blood SCNA-SEQ, it was found that inhibiting APOC1 could reverse the M2 phenotype to the M1 phenotype through the iron death pathway (Hao et al. 2022). Another study by Liu et al. analyzed receptor-ligand pairs in single-cell RNA-seq and demonstrated that SPP1 mediates crosstalk between HCC cells and macrophages through SPP1-CD44 and SPP1-PTger4 associations. In vitro experiments further confirmed that SPP1 can trigger macrophages to polarize into the M2 phenotype TAM (Liu et al. 2022b). External factors can also contribute to TAM polarization. Studies have shown that increased purine metabolism promotes TAM transformation to an end-differentiated phenotype, which leads to decreased efficacy of immune checkpoint blocking (Li et al. 2022). Additionally, inhibition of M2-type macrophage-associated factors can be achieved through the use of specific immune stimulants or immune checkpoint inhibitors. A study in 2023 found that TREM2 + TAM, obtained through ScRNA-seq after TACE treatment, is highly expressed in the tumor microenvironment and associated with poor prognosis. It obstructs CD8 + T cell recruitment through Gal-1 and leads to overexpression of PD-L1. In vitro mouse models demonstrated that knocking out the TREM2 + gene improved the efficacy of anti-PD-L1 (Tan et al. 2023). Similarly, Weng et al. found that PPT1 + TAM was highly infiltrated in liver cancer and led to overexpression of PD-1. The therapeutic effect of anti-PD-1 antibodies was enhanced when PPT1 inhibitors were used (Weng et al. 2023).

By utilizing multi-dimensional single-cell sequencing technology, we can gain insights into the liver tumor microenvironment from the perspective of immune cells. This allows us to uncover the significant role played by liver tumor macrophages in the initiation and progression of tumors. Consequently, we can identify potential therapeutic targets for further exploration.

## Immunotherapy and prognosis of liver tumors

Immunotherapy is a strategy that utilizes the patient's immune system to combat tumors. In liver tumors, the immune escape mechanism, characterized by an increase in immunosuppressive cells and abnormal antigen presentation pathways, hampers the tumor immune surveillance function. By activating the patient's immune system, immunotherapy can enhance the killing effect on tumor cells, thereby effectively treating liver tumors. Liver cancer immunotherapy can be broadly categorized into the following: (1) immune checkpoint inhibitors, such as anti-CTLA-4 antibodies and anti-PD-1/PD-L1 antibodies, which have been extensively studied and shown to significantly improve the prognosis of liver tumor patients; (2) CAR T cell therapy, a treatment that genetically modifies the patient's T cells to recognize tumor-specific antigens and eliminate tumor cells, has demonstrated promising efficacy in clinical trials for liver tumors; (3) Tumor vaccines, which activate the body's immune system to generate an immune response against tumor-specific antigens, have also been investigated and implemented in the treatment of liver tumors.

In the 2023 NCCN Guidelines for liver cancer in the United States, it is proposed that Attilizumab (PD-L1) combined with bevacizumab (VEGF) should be considered as the first-line treatment for advanced unresectable liver cancer. This recommendation is based on the positive results from numerous clinical trials (Casadei-Gardini et al. 2021; Maesaka et al. 2022). The combination of immunotherapy and molecular targeted therapy has been shown to significantly improve the effectiveness of treatment for advanced liver cancer. With the advancement of single-cell multi-omics technology, researchers have been able to identify many new potential therapeutic targets. For instance, in the case of hepatic duct cancer, which is not responsive to immune checkpoint inhibitor therapy (ICB), Yang et al. discovered that PD-L1 + TAM promotes CCA progression and is accompanied by the emergence of bone marrow-derived suppressor cells (G-MDSC) in hepatic duct cancer. These G-MDSCs mediate immune escape by impairing T cell response. However, the simultaneous inhibition of TAM and G-MDSCs enhances the efficacy of ICB. Therefore, targeting G-MDSCs in combination with PD-L1 is similar to the approach of using Attilibead combined with bevacizumab (Loeuillard et al. 2020). Other researchers have also found a significant correlation between PD-L1 expression in liver cancer and the B-cell signature CD20, which leads to reduced infiltration of cytotoxic CD8 + T cells. Thus, a promising treatment strategy is to target B cells in combination with anti-PD-L1 therapy (Feng et al. 2022). Furthermore, single-cell sequencing technology not only helps identify potential therapeutic targets to enhance the effectiveness of ICB but also enables the prediction of patients' responses to immunotherapy. Sun et al. conducted ScRNA-seq and in vivo and in vitro experiments to investigate the impact of GSK3β reduction in TAM on the sensitivity of anti-PD1 immunotherapy for HCC. They discovered that this reduction leads to a decrease in PD-L1 ubiquitination, thereby enhancing the therapy's effectiveness. Additionally, the study revealed that high expression of CD14 + GSK3β + in peripheral blood mononuclear cells (PBMC) can serve as a predictive marker for non-response to anti-PD1 therapy (Sun et al. 2022). Although liver tumor immunotherapy shows promise as a novel therapeutic approach, its success is still influenced by various factors, including the immune characteristics and escape mechanisms of the tumor. Consequently, further research and clinical trials are necessary to determine the optimal immunotherapy strategy and enhance outcomes for patients with liver tumors.

Due to the significant success of CAR-T therapy in treating CD19-positive hematologic malignancies, its application in the treatment of solid tumors like liver cancer has also been explored. In addition to utilizing single-cell technology to identify new targeted antigens, such as Glypican-3 (GPC3), Alpha-fetoprotein (AFP), and EpCAM, CAR-T cell therapy has shown potential as a treatment option for liver cancer when combined with immune checkpoint inhibitors (Chen and E C-Y, Gong Z-W, Liu S, Wang Z-X, Yang Y-S, 2018; Makkouk et al. 2021). Furthermore, gene editing can be employed to enhance the durability and anti-tumor activity of CAR T cells. Recent research has focused on personalized vaccines, which are tailored to a patient's specific tumor characteristics to enhance the immune response. By analyzing the genomic and epigenetic features of tumors, researchers can identify suitable antigens for personalized vaccines, thereby improving their efficacy (Zongyi and Xiaowu 2020). Next-generation sequencing techniques can also be utilized to identify potential neoantigens and design vaccines by comparing genomic and proteomic differences between normal and cancerous liver tissue in HCC patients. However, there are still challenges to overcome in the use of therapeutic vaccines for HCC, such as the immunosuppressive tumor microenvironment that induces antigen-specific T cell tolerance, leading to reduced vaccine effectiveness (Zhou et al. 2021).

The current treatment of liver cancer relies on comprehensive treatment programs, including surgery, chemotherapy, transarterial chemoembolization (TACE), and immunotherapy. With the advancement of single-cell multi-omics technology, we now can understand the cellular interaction in the immune microenvironment of liver tumors and explore various pathways of cellular chemokines. This technology also allows us to identify potential new therapeutic targets and develop precision medicine approaches. By integrating different treatment strategies, we can enhance their effectiveness and achieve the goal of precision medicine.

### Advantages and limitations

This article presents the first bibliometrics study on the application of single-cell sequencing in liver cancer research. Bibliometrics is a comprehensive and objective method for literature research, which can reveal the research history, trends, and hot topics in a specific field. It provides a logical map for researchers in the field, based on related studies (Wu et al. 2022a; Tao et al. 2022; Zhu et al. 2022). Our research also demonstrates these advantages. To minimize bias introduced by research software and technologies, we have taken two measures. Firstly, we utilized recognized tools such as VOSviewer, Citespace, and the R package 'bibliometrix' for comprehensive analysis (Pan et al. 2018). Secondly, we constructed cluster analysis, time map, and peak map, and further developed a time zone map to showcase the origins and specific directions of hot frontier trends, while mitigating the impact of clustering.

Our study acknowledges the limitations commonly found in bibliometric articles (Ogunsakin et al. 2022; Sheng et al. 2021). While we utilized the WOSCC database, which has a well-established citation network (Birkle et al. 2020), it is important to note that not all studies in the field may be included.

## Conclusion

Liver cancer is a serious global health threat, and understanding its occurrence and development is crucial. Single cell sequencing is invaluable for analyzing tumor heterogeneity, describing the tumor microenvironment, and identifying potential immunotherapy targets and strategies for liver cancer. In this bibliometric research article, we aim to explore the history, evolution, and current frontiers of single-cell sequencing in liver cancer research. We present visual analysis results based on countries, institutions, and authors to examine the development and collaboration in this field. Additionally, we analyze the research potential, influence, and main research directions through journal distribution and co-cited journal networks. Furthermore, we conduct an in-depth analysis of research trends, frontier hotspots, and research directions based on co-cited literature, explosive citations, and keywords. It is worth noting that advancements in single-cell sequencing technologies have enabled us to understand the composition of liver intrinsic cells, macrophages (Kupfer cells), and the components of the liver tumor immune microenvironment, as well as their potential impact on prognosis and immunotherapy. However, the clinical translation of liver cancer therapy guided by single cell sequencing technology remains a critical consideration.

## Supplementary Information

Below is the link to the electronic supplementary material.

Supplementary file1 (XLSX 1358 KB)

## Data Availability

No datasets were generated or analysed during the current study.
